# Protocol for a mixed-methods realist evaluation of a health service user feedback system in Bangladesh

**DOI:** 10.1136/bmjopen-2017-017743

**Published:** 2017-07-05

**Authors:** Bassey Ebenso, Rumana Huque, Zunayed Azdi, Helen Elsey, Shammi Nasreen, Tolib Mirzoev

**Affiliations:** 1 Nuffield Centre for International Health and Development, University of Leeds, Leeds, UK; 2 ARK Foundation, Dhaka, Bangladesh; 3 Department of Economics, University of Dhaka, Dhaka, Bangladesh

**Keywords:** realist evaluation, service user feedback, bangladesh, mixed methods, health systems responsiveness

## Abstract

**Introduction:**

Responsiveness to service users’ views is a widely recognised objective of health systems. A key component of responsive health systems is effective interaction between users and service providers. Despite a growing literature on patient feedback from high-income settings, less is known about effectiveness of such systems in low-income and middle-income countries.

**Methodology and analysis:**

This paper disseminates the protocol for an 18-month ‘RESPOND’ project that aims to evaluate the system of collecting and responding to user feedback in Bangladesh. This mixed-method study uses a realist evaluation approach to examine user feedback systems at two Upazila health complexes in Comilla District of Bangladesh, and comprises three steps: (1) initial theory development; (2) theory validation; and (3) theory refinement and development of lessons learnt. The project also uses (1) process evaluation to understand *causal mechanisms* and *contexts of implementation*; (2) statistical analysis of patient feedback to clarify the nature of issues reported; (3) social science methods to illuminate feedback processes and user and provider experiences; and (4) health policy and systems research to clarify issues related to integration of feedback systems with quality assurance and human resource management. During data analysis, qualitative and quantitative findings will be integrated throughout to help achieve study objectives. Analysis of qualitative and quantitative data will be done using a convergent mixed-methods model, involving continuous triangulation of multiple data sets to facilitate greater understanding of the context of user feedback systems including the links with relevant policies, practices and programmes.

**Ethics and dissemination:**

Ethics approvals were obtained from the University of Leeds and the Bangladesh Medical Research Council. All data collected for this study will be anonymised, and identifying characteristics of respondents will not appear in a final manuscript or reports. The study findings will be presented at scientific conferences and published in peer-reviewed journals.

**Strengths and limitations of this study**

- Adopting a structured realist methodology will shed light on how the *Context* of implementation of user feedback affects intervention *Mechanisms* (eg, actors’ behaviour in implementing systems) to produce intended and unintended *Outcomes.*
- The gaps in literature on user feedback, combined with a focus on practical issues raised by policymakers and funders, create a favourable environment for the study to generate new knowledge.
- Involving health managers and decision-makers in designing and assessing performance of user feedback systems can foster ownership of the results and ensure sustainability of interventions to improve health systems responsiveness in Bangladesh.
- Managing various disciplines in the study design (ie, process evaluation, statistical analysis, social science methods and health policy and systems research) can be a challenge.
- Implementing the study in only one district may affect generalisability of study findings to the whole country.

## Introduction

Responsiveness to service user views is a widely recognised objective of national health systems.^1–3^ Responsive health systems anticipate and adapt to future health needs, and harness emerging opportunities to promote universal access to effective interventions.^4^ A key component of responsive health systems is effective interaction between service users and service providers or managers.^5–8^ This interaction is important in two ways. First, it gives service users the opportunity to provide feedback on issues such as their experiences of the care they received, perception of staff expertise, availability of supplies and so on.^8–11^ We use the term ‘feedback’ as this includes both complaints (ie, grievances) and praises (ie, positive reflections) from service users. Second, the interaction provides the health system with the opportunity to collect, respond to and use user feedback in, for example, improving health service quality^12 13^ or strengthening human resource (HR) management processes.^13–15^ There are two approaches to collecting user feedback: one in which data collection is led by service providers, researchers or managers (eg, using surveys, critical incident techniques, case studies and interviews),^10^ and the other in which service users actively provide information (eg, through suggestions boxes and telephone hotlines).^16^ The RESPOND project focuses on user-initiated feedback.^17 18^

Health service users, or patients, across the world are increasingly asked to voice their opinions about service development and/or provide feedback on their experiences of healthcare services.^19^ A systematic review of the impact of involving users in healthcare found evidence of improved health services.^20^ However, much of this evidence is from high-income countries, highlighting limited research on patient involvement in healthcare in low-income and middle-income countries (LMICs). Another gap identified from recent reviews is the need to develop effective grievance redressal systems that ensure that patient feedback is responded to^21^ and acted upon,^13 22 23^ because patients who do not receive responses to their feedback (especially complaints) are more likely to feel frustrated and disengaged with health services.

Health programmes (including patient feedback systems) are inherently complex, and their success is determined by how the programmes are implemented within the wider health system’s context. Theory-driven forms of evaluation help in understanding such complexity by studying how different elements of interventions are intertwined^24^ and recognising the role of context as a key influence in the production of outcomes.^25^ Realist evaluation (RE) is a theory-driven evaluation approach that is increasingly used for studying the implementation of complex interventions within health systems, including in LMICs.^26 27^ A realist approach emphasises the contingent nature of programme outcomes and addresses questions about what works, in which setting, for whom, in what circumstances and why.^25^ In RE, researchers develop *middle-range theories* (MRTs) that take account of how *Context* (at micro, meso and macro levels) influences intervention processes or *Mechanisms* (eg, actors’ behaviours in implementing intervention) to produce intended and unintended *Outcomes*. This is known as a C-M-O configuration,^25^ and CMOs allow accounting for all these dimensions, ensuring that all key aspects of the programme are recorded, thus helping to maintain the validity and reliability of results.^25^

The Ministry of Health and Family Welfare (MOHFW) in Bangladesh strives to improve the health and well-being of vulnerable people (eg, women and the poor), earmarking >60% of its health spending to the Essential Service Package, a major share of which is spent at the Upazila health complex (UHC) level.^19^ The UHC is the backbone of Bangladesh’s health system as UHCs are the first-level referral services from the primary healthcare facilities (community clinics, union health and family welfare centres). A UHC serves a population of 200 000–400 000 people. Each UHC has a health management committee that monitors service provision, and identifies and addresses emerging problems at UHCs. Membership of UHC management committee includes local politicians, health facility managers, civil society representatives and community leaders.

Since 2009, the MOHFW has implemented a national programme to enhance service users’ voice through allowing them to provide feedback on their experiences of using health services in Bangladesh using a short messaging service (SMS), in addition to a more traditional use of suggestion boxes in health facilities.^28 29^ All SMS texts go into a publicly available national web portal (http://app.dghs.gov.bd/complaintbox/?actn=lstmsg), with many entries containing issues that service users provided feedback on, dates of receipt of feedback and of solution to issues raised by users. This SMS feedback system is monitored by the MOHFW. Each SMS is subsequently followed up with a phone call to the sender and the local authority of the health facility that the feedback was about. Additional to the SMS feedback and suggestion boxes in each health facility, service users can send feedback directly to UHC management committees at the subdistricts. However, it is unclear who is responsible for following up issues fed back to grassroots-level health facilities (such as UHCs) and how this follow-up is done. It is equally unclear how well the systems of collecting and responding to feedback are integrated with and/or used for supporting staff supervision and performance appraisal and service quality assurance at the Upazila level.

Furthermore, national policymakers recognise that the implementation of the user feedback programme is patchy and needs strengthening. For example, while the MOHFW receives approximately 1000 messages per day, it has only two dedicated staff to follow up SMS feedback. No information is available as to the type of feedback received directly by the health management committees and to what degree the issues are addressed. As a result, ensuring responsive health system in one of the world’s most densely populated countries remains a major challenge.

The purpose of this paper is to share the study protocol for an RE of service user feedback programme in Bangladesh. This paper should be of interest to researchers interested in methodologies for assessing procedures for collecting and addressing service user feedback, as well as to policymakers and practitioners interested in designing or evaluating interventions for improving the responsiveness of user feedback systems as part of improving quality of service.

This study aims to better understand the processes of and environment within which service users’ feedback is collected, in order to assist policymakers design a comprehensive health systems intervention to make the health system in Bangladesh more responsive to patient feedback. The specific project objectives are to work closely with national and local decision-makers to:

1. develop an in-depth understanding of the nature, contents of and key reasons why patients provide feedback to health services at district and subdistrict (Upazila) levels
2. analyse the processes of collecting and responding to service users’ feedback at Upazila level, and the key contextual facilitators and constraints influencing these processes
3. assess the approach to and processes of service quality assurance and HR management, focusing on the use of feedback from service users at Upazila level
4. using results of objectives 1–3 to develop a comprehensive and context-specific health systems intervention to improve the use of feedback from service users in quality assurance and HR management processes at Upazila level.

The achievement of project objectives is a crucial first step of a longer term plan to implement and assess a comprehensive intervention at larger scale to improve responsiveness of the health system in Bangladesh. The close links between the MOHFW and our project partners in Bangladesh (ie, ARK Foundation) will facilitate scaling up and developing the national policy to make the country’s health system more responsive through better integrating user feedback within quality assurance and HR management.

There are no widely used systems for disseminating RE protocols. Therefore in reporting our study protocol, we draw on different checklists for reporting empirical results. These include COnsolidated criteria for REporting Qualitative research (COREQ) standards for reporting qualitative research^30^ and a recently published Realist And Meta-narrative Evidence Syntheses: Evolving Standards (RAMESES) II reporting standards for REs.^31 32^ In this protocol, we outline the study design and methods including study setting, conceptual framework, data collection and analysis methods. We also explain researchers’ background, key ethics and research governance issues, and our approach to dissemination.

## Study design and methods

### Study setting and target population

The 18-month study (January 2017–June 2018) will be implemented in Comilla District, which lies south-east of the capital, Dhaka. This district was selected in consultation and discussion with the MOHFW, based on the district receiving frequent user feedback, the existence of a motivated district health leadership and our previous successful experience of working within the context of Bangladesh. We will purposefully select two UHCs in Comilla following an initial review of the feedback environment in the district using non-participant observation and review of publicly available documents. One UHC will have a ‘favourable’ feedback environment (ie, clear signs explaining where and how to provide feedback and the processes for responding to service user feedback), and the other UHC will have a ‘less favourable’ feedback environment (ie, no clear signs or processes for dealing with feedback).

Target populations for the study are (1) health service users at UHC, most of whom are groups such as women or poor people, (2) service providers and managers, and (3) health planners and policymakers. The intervention, to be designed in achieving objective 4, will include detailed guidance for each target group. For service users, the intervention will detail methods for enhancing their engagement with feedback systems (eg, improving awareness of strengths of current systems, using health management committees). For providers, managers and policymakers, we will have context-specific tools for improving utilisation of user feedback in quality assurance (eg, critical incident technique) and HR management (eg, revised supervision format, contents of staff performance reviews).

### Conceptual framework

**Figure 1:**
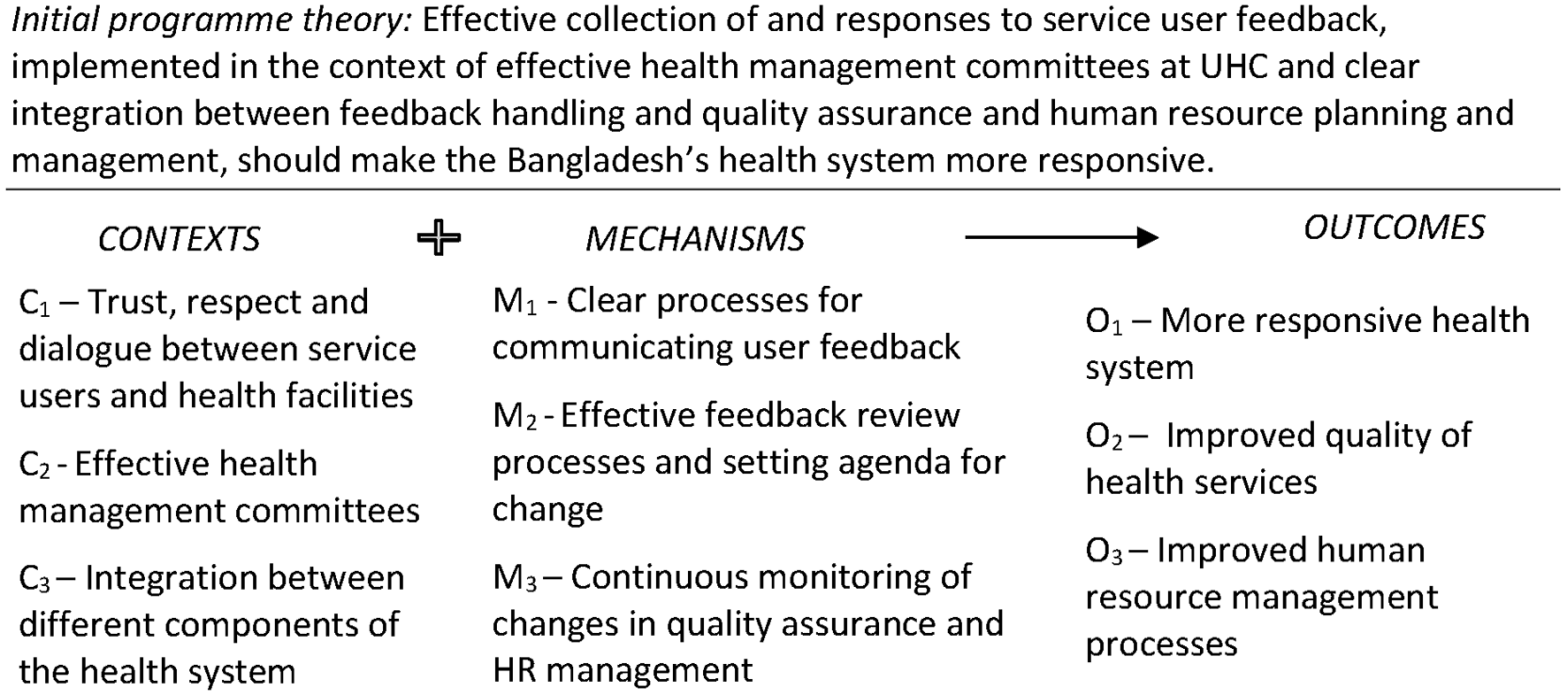
Conceptual framework for study. HR, human resource; UHC, Upazila health complex.

This is designed as a *multidisciplinary and mixed-method study* that uses RE to examine user feedback systems in Bangladesh. The RE approach helps to address questions about what works, for whom, in which circumstances and why. Researchers use the approach to empirically develop, validate and refine MRTs that account for how the *Context* in which interventions are implemented influence intervention *Mechanisms* (eg, actors’ behaviour in implementing intervention) to produce intended and unintended *Outcomes*. As mentioned earlier, this is known as a C-M-O configuration. Figure 1 summarises the initial programme theory, which will be continuously validated and refined during data collection and analysis. Detailed C-M-O configurations (eg, C_1_+M_2_=O_2_) will be developed and will include specific Cs, Ms and Os (identified through objectives 1–3). These, together, will inform the design of the comprehensive health systems intervention in pursuit of the project’s objective 4.

REs are method-neutral,^25^ meaning that researchers can use combinations of methods from different disciplines for the evaluation.^33^ In line with this, we will draw upon process evaluations, statistics, social sciences and health policy and systems research (HPSR), although HPSR can be seen as encompassing all these different disciplines.^34^ Process evaluation will be used to clarify**causal mechanisms** and *context of implementation of user feedback systems*.^35^ Analysis of statistical data from the government web portal and UHC facility records will help clarify the nature of issues reported and their distribution by timing, gender, age or location. Social science methods will guide in-depth analysis of feedback processes, and user and provider experiences of feedback systems. HPSR will help analyse the integration of feedback systems with quality assurance mechanisms and HR management.

### Methods of data collection and sampling

The study will be implemented in the following three phases:

1. initial theory development: methodology development including developing initial working theories for the patient feedback system (phase 1)
2. theory validation and refinement, using continuous rounds of data collection and analysis (phase 2)
3. theory consolidation and developing a comprehensive intervention (phase 3).

Due to the evolving and incremental nature of realist studies, some methodological elements of data collection and analysis will be developed at later stages.

#### Phase 1: initial theory development

Phase 1 has already started, and included three activities and covered the first 2–3 months of the study. First, the research team (1) reviewed publicly available documents related to the service user feedback systems, (2) visited UHCs to conduct non-participant observations of the complaints environment and (3) held informal discussions with 3–5 key stakeholders (such as facility-in-charge and policymakers) to develop and refine programme theories, that is, hypothetical pathways that help explain feedback system(s) and link it/them to existing systems of quality assurance and HR management within the context of Comilla District, Bangladesh. These initial engagements were informal and did not constitute formal interviews requiring ethics clearance (eg, discussions were not audio-recorded, although stakeholders were made aware of the study using information from relevant participant information sheets). The discussions were organised primarily to facilitate buy-in and project ownership from relevant policymakers and programme implementers. Second, the teams in Leeds and in Bangladesh obtained ethics approvals from the University of Leeds and the Bangladesh Medical Research Council, respectively. Third, we will develop data collection tools, based on the initial programme theories to be used in phase 2 of the study.

At the end of this phase, the detailed programme theories will be developed to help us explore the relations between context, processes (or mechanisms) and outcomes (also called C-M-O configurations) of the feedback system further in the project.

#### Phase 2: theory validation and refinement

Phase 2 is scheduled to start from the second quarter of year 1 and last for about 8–10 months, and will include adaptation and pilot testing of generic qualitative and quantitative data collection tools, followed by the bulk of fieldwork to validate and refine programme theories.

We will use different qualitative and quantitative methods to understand and validate programme theories that link context, mechanisms and outcomes of user feedback system. These will be updated, reflecting the evolution of specific MRTs, and will include combinations of the following:

1. In-depth interviews (IDIs) with service users (about 20 in each UHC, which in our experience is sufficient to capture key perspectives and achieve data saturation) and focus group discussions (FGDs) with community members (2–3 FGDs in each UHC) to explore their knowledge and use of feedback systems. Participants will be purposively selected based on gender, age and use of feedback systems.
2. IDIs with purposefully selected service providers and managers (about 10 in each UHC, which in our experience is sufficient to capture key perspectives and achieve data saturation) to explore their views on and experience engaging with the user feedback systems.
3. Analysis of country-level secondary data on user feedback from the web portal and UHC records to understand types of issues, location, gender and age of users who initiated issues.
4. Non-participant observation of feedback environment in the subdistrict, health management committee meetings and UHC routine quality assurance and staff management practices.
5. Review of key documents, for example, feedback to users and actions taken, meeting minutes, quality assurance guidelines, staff performance appraisal and supervision records.

The respondents for IDIs, identified through purposive sampling, will include UHC managers, health staff, health planners/policymakers at subdistrict, district and national levels and selected service users. A detailed list of respondents will be developed after phase 1, and snowballing technique will be used to identify any further informants. We will aim to conduct two to three focus groups with community members in each UHC to explore their knowledge and use of feedback systems. However, if we reach data saturation earlier (ie, when further respondents do not reveal new themes for analysis), these numbers of IDIs and FGDs may decrease in order not to collect any unnecessary data. Different experiences of the user feedback system are expected to emerge within different subgroups of respondents. So both men and women and all age groups in the above subgroups are considered for inclusion (18–65 years).

The detailed interview and focus group question guides will be developed to inform fieldwork during phase 2. These will be informed by the study conceptual framework and structured around the study research objectives to explore programme theories developed during phase 1. Question guides will be adapted to the different groups of stakeholders, commensurate to their backgrounds, degree of involvement and particular roles in the design and implementation of the patient feedback system.

### Data analysis

All interviews will be audio-recorded (subject to informed consent), transcribed and where appropriate translated into English for analysis. Framework approach will be used to test hypotheses, while allowing for emergence of new themes, and will include stages of familiarisation, coding, indexing and charting, mapping and interpretation.^36^ The qualitative and quantitative data collection methods will be integrated throughout, and their combinations will be required to achieve the project objectives. Analysis of qualitative and quantitative data will be done using convergent mixed-methods model, that is, involving continuous triangulation of multiple data sets,^37^ and enable greater understanding of the context of user feedback system, including the links with relevant policies, practices and programmes. We will share interpretations and summary findings of our analysis with key stakeholders. This is discussed below as part of the first workshop of phase 3.

#### Phase 3: theory consolidation and developing a comprehensive intervention

During this phase, we will summarise our refined programme theories in form of MRTs to help articulate a theoretically robust and empirically tested model of complex relations between the contexts, mechanisms and outcomes of patient feedback system in Bangladesh.

**Figure 2:**
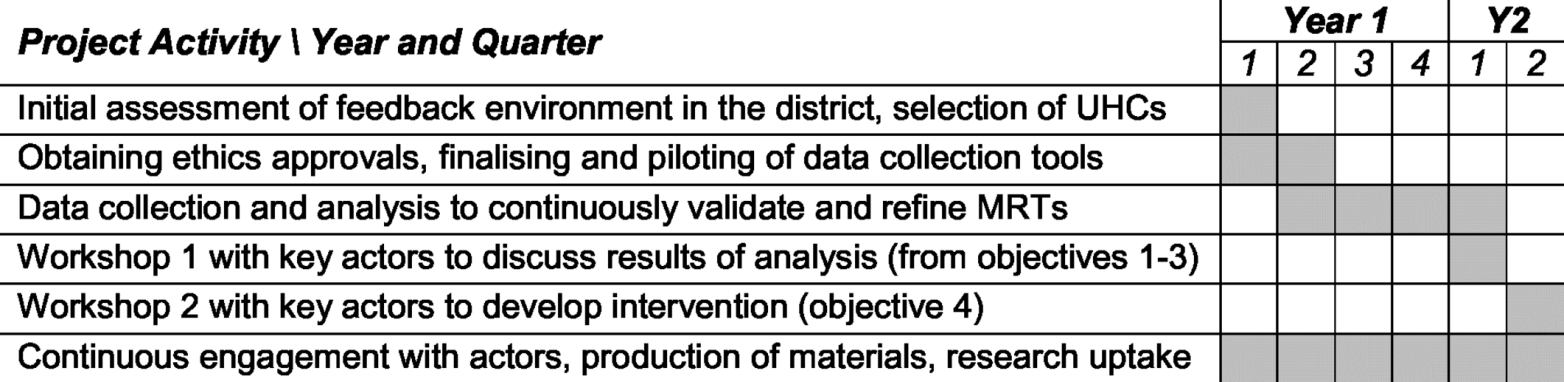
Project work plan. MRT, middle-range theories; UHC, Upazila health complex.

We will also use this phase to develop a comprehensive health systems intervention to improve utilisation of user feedback in health service quality assurance and HR management processes at Upazila level. As part of this, we plan to have two workshops with key stakeholders. The first workshop will be *used to share and discuss results of our analyses with key stakeholders* for comment and correction, to ensure our interpretations and conclusions match participants’ beliefs and experiences of user feedback system. During the second workshop, we will facilitate *development of a comprehensive health systems intervention by the key health policy actors* in Bangladesh. A project work plan is shown in figure 2.

As shown in figure 2, there are some overlaps among the three project phases. We do not see the progression between the different phases as a linear process: that is, as part of the analysis, we are likely to identify further hypothetical pathways that may require further data collection and analysis. The specific programme theories that emerge will be continuously refined and will provide a framework for the data collection and analysis, in line with the principles of RE. We will work with decision-makers in a research–policy partnership^38^ to facilitate adoption of results into policy.

### Researchers’ background

The research team comprises three men (BE, TM, ZA) and three women (HE, RH, SN). BE is a research fellow in HPSR at the University of Leeds, UK, and has expertise in methodology development for mixed-methods evaluation of complex applied healthcare interventions. RH is a professor of health economics at the University of Dhaka, Bangladesh. She has a background in health systems strengthening. TM is an associate professor of HPSR, while HE is associate professor of public health at the University of Leeds, and they are both experienced in applying RE methodologies. ZA and SN both hold Master’s of Public Health and serve as research staff at the ARK Foundation in Dhaka, Bangladesh. They apply qualitative and RE methods to studies of maternal and child health in Bangladesh.

The qualitative interviews will be conducted by ZA and SN, who are trained and experienced in conducting and analysing data from patients, providers and policymakers in the context of healthcare provision in Bangladesh. They have no direct connections to the participants or study sites in Comilla District as their qualitative interview training and experiences (including in RE interviews) were acquired in other districts of Bangladesh, so their distance from participants and studying the sites should constitute a strength. However, as Stanley and Nayar^39^ recommend a reflexive approach to managing the researcher–participant relationships, ZA and SN will maintain a research journal that captures their experiences of researcher–participant relationship with study respondents, as a way to ensue study rigour. The journal will explain the reasoning behind decisions made during data analysis and include the pros and cons of a distant relationship with research participants on eliciting research data that are a true reflection of participants’ beliefs.

### Ethics and research governance

Ethical approvals for this study were obtained from the School of Medicine Research Ethics Committee at the Faculty of Medicine and Health at the University of Leeds (ref: MREC16-110) and the Bangladesh Medical Research Council (ref: BMRC/NREC/2016-2019/164). These are available in online supplementary files.

The project will be carried out with full respect for current relevant legislations (eg, the Charter of Fundamental Rights of the European Union) and international conventions (eg, Helsinki Declaration). The methods development, data collection and analysis will take account of the following issues:

- *Anonymity* of study respondents will be preserved where possible and will be ensured at all times if respondent(s) requests. Unnecessary collection of personal data will be avoided and respondents will have the right to review study outputs and withdraw consent if necessary. Where personal data are collected, it will be coded, removed from the data for analysis and stored separately from transcripts. Only Principal Investigators (PIs) and designated research personnel in each partner institution will have access to the keys linking the data with the personal information.
- *Informed consent* will be obtained from all study participants, and in the case of refusal alternative means of data collection will be explored (eg, alternative respondents).
- Specific emphasis will be placed on *confidentiality and other data protection issues*, which will include security of data storage and access rights to data. Only members of teams identified by the PIs in each institution will have access to the data. Where project data (eg, interview transcripts) are stored on an institutional server, it will be password-protected and only members of the research team will have access to the passwords. The availability of documents on the internet will be with the consent of both project partners.

The project will be implemented according to standard governance practice at the University of Leeds for the implementation of collaborative projects. This includes ensuring regular communication between the partners and engagement with policymakers and practitioners; quality assurance through regular peer review both within and between the teams; appropriate mentoring and coaching support of junior researchers; and ensuring equal opportunities to both genders.

### Communication and dissemination of results

Adequate communication of results to inform policy and practice is an essential component of any health systems and policy research. We will ‘embed’ the study into policy and practice, working with national, regional and local actors. This approach, developed by the Nuffield Centre, has been effective in many countries in improving the quality and effectiveness of the implemented programme.^40^ Decision-makers at district and MOHFW levels will be continuously engaged throughout the process in a research-policy partnership to facilitate adoption of effective strategies and tools.^38^ The study results will be used — through the ‘embedded’ research and development approach within policymaking and programme planning — to contribute further improvements in healthcare provision and achievement of better health outcomes. Specific methods of communicating research findings will include combinations of the following:

1. delivering presentations at review meetings at district and national levels in Bangladesh (eg, semiannual and annual reviews involving national and international policy actors)
2. developing newsletters and press releases aimed at communicating key study findings in ways that are accessible to the general public in Bangladesh and wider within Asia
3. developing policy briefs addressed to national and international policymakers and practitioners and designed as short and practical documents
4. possible interviews in the national media (eg, radio and television) as well as articles for national newspapers, communicating our findings and educating the public as needed
5. developing a dedicated website for the project where the project results will be publicly accessible by national and international decision-makers, practitioners and academics
6. delivering presentations at national, regional and international conferences and publication of articles in peer-reviewed journals with specific emphasis on open access where feasible
7. developing a project research report for the funder, with a publishable executive summary.

Building responsive health systems is a priority, both nationally and internationally. The existence of an ongoing government programme to enhance users’ voice, a clear intention to further strengthen the programme and a strong interest for this research from the national and local decision-makers in Bangladesh provide an excellent opportunity to generate high-quality evidence and ensure its highest impact on policy and practice in Bangladesh.

## Discussion

In this paper, we have reported a study protocol for RE of patient feedback system in the context of Bangladesh. This is designed as a multidisciplinary and mixed-methods research that aims to better understand the system of patient feedback, in order to assist policymakers design a comprehensive health systems intervention to make the health system in Bangladesh more responsive.

Since the start of the study, three initial hypothetical pathways or initial working theories (IWTs) have been developed at a workshop held in February–March 2017. The first initial working theory (IWT1) focuses on the motivation for and willingness of service users to provide feedback, the second (IWT2) deals with processing and analysis of complaints at UHCs, while the third (IWT3) focuses on acting on user feedback and providing users with relevant feedback for their complaints. These IWTs (which progressed from the overall programme theory shown in figure 1) are currently being further developed using literature review, analysis of key documents and limited number of interviews with key stakeholders as part of the project’s phase 1. Each IWT (IWT1, IWT2 and IWT3) identified specific Cs, Ms and Os developed from researchers’ understanding of the user feedback programme, informal engagements with key MOHFW personnel and UHC staff in Bangladesh and review of relevant literature on the subject. The relationships between and among these specific Cs, Ms and Os will be explored as part of data collection for the study. The IWTs were subsequently translated into the specific information areas to guide the development of specific tools for primary data collection.

The following aspects of the context within which this study is implemented are worth mentioning. First, the health systems environment in Bangladesh currently promotes evidence-informed health policymaking, as we found within our previous collaborative projects. The commitment by key health decision-makers at Comilla District and the national MOHFW to engage with this study is a particular strength of this project. This will ensure the high ownership and potential for impact of the comprehensive intervention to be developed by the key stakeholders during the later stages of the project. Second, at theoretical level, the gaps in the literature on user feedback, combined with an increasing interest in applied research focusing on practical issues raised by policymakers and funders, create a favourable environment for the study to generate new knowledge. The study findings will provide a timely contribution to an ongoing debate about processes for and effectiveness of user feedback systems in LMICs.

This study has a potential to improve understanding of the functioning of user feedback system in Bangladesh, including in-depth understanding of key contextual factors at macro, meso and micro levels affecting this performance. The study results can be used to achieve improvements in policymaking, health systems strengthening and improvements in health outcomes. In line with this, specific impacts of our study on policy and practice in Bangladesh and internationally include the following:

1. improvements in user feedback systems, implemented to empower the public to hold health system to account and enhance the responsiveness of Bangladesh’s health system
2. developing local expertise on the design and implementation of context-specific health systems interventions to ensure user feedback is processed and acted upon
3. utilisation of innovative, cross-disciplinary, approaches for assessing effectiveness of complex interventions including user feedback systems
4. scientific advancement of theories on how to make health systems more responsive in the context of LMICs.

This study will make a vital contribution to health systems’ responsiveness in Comilla District and more widely across Bangladesh. Evaluation of complex interventions such as service user feedback systems and their longer term impact on quality assurance and HR management requires a comprehensive understanding of intervention context, implementation, mechanisms and outcomes. The multidisciplinary and mixed-method realist approach that this study adopts will facilitate such evaluation

## Supplementary Material

Reviewer comments

Author's manuscript
