# Supplementary material for: Protocol for a mixed-methods realist evaluation of a health service user feedback system in Bangladesh
Source: BMJ Open. 2017 Jul 5;7(6):e017743. doi: 10.1136/bmjopen-2017-017743 (PMC5734574; doi:10.1136/bmjopen-2017-017743)
Supplement: Author's manuscript [file bmjopen-2017-017743.draft_revisions.pdf]

BMJ Open

Study protocol: Realist evaluation of health service user feedback system in Bangladesh

Journal:	*BMJ Open*
Manuscript ID	bmjopen-2017-017743
Article Type:	Protocol
Date Submitted by the Author:	14-May-2017
Complete List of Authors:	Ebenso, Bassey; University of Leeds Faculty of Medicine and Health, Nuffield Centre for International Health and Development<br>Huque, Rumana; ARK FOUNDATION , Road 21, House B130, New DOHS; University of Dhaka Department of Economics, Department of Economics<br>Azdi, Zunayed; ARK FOUNDATION , Road 21, House B130, New DOHS<br>Elsey, Helen; University of Leeds, Nuffield Centre for International Health and Development<br>Nasreen, Shammi; ARK FOUNDATION , Road 21, House B130, New DOHS<br>Mirzoev, Tolib; University of Leeds, Nuffield Centre for International Health and Development
**Primary Subject Heading**:	Patient-centred medicine
Secondary Subject Heading:	Health policy, Health services research, Global health
Keywords:	Realist evaluation, Service User Feedback, Bangladesh, mixed methods, health systems responsiveness

SCHOLARONE™ Manuscripts

For peer review only - http://bmjopen.bmj.com/site/about/guidelines.xhtml

Word count: 4,026 (this excludes, title page, abstract, references, figures and tables)

Title Page

Study protocol: Realist evaluation of health service user feedback system in Bangladesh.

AUTHORS:

- Bassey Ebenso*[1] B.E.Ebenso@leeds.ac.uk
- Rumana Huque[2,3] rumana@arkfoundationbd.org
- Zunayed Azdi[2] azdi@arkfoundationbd.org
- Helen Elsey[1] H.Elsey@leeds.ac.uk
- Shammi Nasreen[2] shammi@arkfoundationbd.org
- Tolib Mirzoev[1] T.mirzoev@leeds.ac.uk

**Corresponding author*

Bassey Ebenso
Nuffield Centre for International Health and Development,
Leeds Institute of Health Sciences
University of Leeds,
Room 10.28, Worsely Building
Clarendon Way,
LS2 9NL
Leeds, UK.

Email: B.E.Ebenso@leeds.ac.uk

Phone: +44 (0)1133439642

Authors institutional addresses:

[1] Nuffield Centre for International Health and Development, University of Leeds, Worsely Building, Clarendon Way, Leeds, UK.
[2] ARK Foundation, Road 21, House B130, New DOHS, Mohakhali, Dhaka, Bangladesh
[3] Department of Economics, University of Dhaka, Dhaka, Bangladesh

KEY WORDS:
Realist evaluation, service user feedback, Bangladesh, mixed methods, health systems responsiveness

For peer review only

For peer review only - http://bmjopen.bmj.com/site/about/guidelines.xhtml

Word count: 4,026 (this excludes, title page, abstract, references, figures and tables)

ABSTRACT

Introduction
Responsiveness to service users' views is a widely-recognised objective of health systems. A key component of responsive health systems is effective interaction between users and service providers. Evidence shows that policymakers and managers are increasingly interested in collecting and analysing user feedback as this can inform service improvements and health outcomes. However, despite a growing literature on patient feedback from high-income settings, less is known about effectiveness of such systems in low and middle income countries (LMICs).

Methodology and analysis
This paper disseminates the protocol for an 18-month 'RESPOND' project that aims to evaluate the system of collecting and responding to user feedback in Bangladesh.

This mixed-method study uses a Realist Evaluation approach to examine user feedback systems at two Upazila Health Complexes in Comilla district of Bangladesh, and comprises three steps: i) initial theory development; ii) theory validation; and iii) theory refinement and development of lessons learned. The project will utilise: i) Process evaluation to understand *causal mechanisms* and *contexts of implementation;* ii) Statistical analysis of quantitative data from patient feedback to clarify the nature of feedback issues; iii) Social science methods to illuminate feedback processes and explore user and provider experiences; and iv) Health policy and systems research to clarify issues related to integration of feedback systems with quality assurance and human resource management.

Ethics approvals were obtained from the University of Leeds and the Bangladesh Medical Research Council.

Discussion
The project will improve understanding of the determinants of performance of user feedback systems in Bangladesh. The study will also assist policymakers design targeted interventions to increase the use of patient feedback for improving health systems responsiveness. Dissemination of this protocol has the potential to stimulate discussion on the use of realist methodologies for assessing user feedback systems as part of quality improvement strategies in LMICs.

KEY WORDS:
Realist evaluation, service user feedback, Bangladesh, mixed methods, health systems responsiveness

For peer review only - http://bmjopen.bmj.com/site/about/guidelines.xhtml

Word count: 4,026 (this excludes, title page, abstract, references, figures and tables)

Article Summary

Article focus

- More research is needed to clarify the processes of implementing, and performance of user feedback systems in low and middle income countries (LMICs). Also needed is a greater understanding of the complexity of health systems and wider environments within which user feedback systems are implemented.
- Dissemination of this study protocol is intended to generate interest in the RESPOND project and, more importantly, stimulate discussion on the use of realist methods for assessing user feedback systems within complex health systems in LMICs.

Key messages

- Using a realist evaluation approach to assess user feedback systems should shed light on how health workers collect, analyse and respond to patient feedback at district level.
- Involving health managers and decision-makers in designing and evaluating performance of existing user feedback systems should foster ownership of the results and promote the sustainability of interventions to improve health systems responsiveness in Bangladesh.

Strength and limitation of this study

- Adopting a structured realist methodology and multi-disciplinary approach is a strength that should facilitate the identification of how the *Context* in which interventions are implemented affect intervention *Mechanisms* (e.g. actors' behaviour in implementing intervention) to produce intended and unintended *Outcomes.*
- The small scale of the study (implemented in one district), may affect generalizability of findings to the whole country. Nonetheless, the realist evaluation approach should improve understanding of the contextual determinants of success of the user feedback system, and by so doing, facilitate generalizability of results.

For peer review only

For peer review only - http://bmjopen.bmj.com/site/about/guidelines.xhtml

Word count: 4,026 (this excludes, title page, abstract, references, figures and tables)

Introduction

In this paper, we report the study protocol for a realist evaluation of service user feedback in the context of Bangladesh (RESPOND project).

Responsiveness to service user views is a widely-recognised objective of national health systems[1-3]. Responsive health systems anticipate and adapt to future health needs, and harness emerging opportunities to promote universal access to effective interventions[4]. A key component of responsive health systems is effective interaction between service users and service providers or managers[5-8]. This interaction is important in two ways. First, it gives service users the opportunity to provide feedback on issues such as their experiences of the care they received, perception of staff expertise, availability of supplies etc.[8-11]. We use the term 'feedback' as this includes both complaints (i.e. grievances) and praises (i.e. positive reflections), from service users. Second, the interaction provides the health system with the opportunity to collect, respond to, and utilise, user feedback in e.g. improving health service quality[12, 13] or strengthening human resource (HR) management processes[13-15]. There are two approaches to collecting user feedback: one in which data collection is led by service providers, researchers or managers (e.g. using surveys, critical incident techniques, case studies and interviews)[10] and the other, in which service users actively provide information (e.g. through suggestions boxes and telephone hotlines)[16]. The RESPOND project focuses on user-initiated feedback.[17, 18]

Health service users, or patients, across the world are increasingly asked to voice their opinions about service development and/or provide feedback on their experiences of health care services[19]. A systematic review of the impact of involving users in healthcare found evidence of improved health services[20]. However, much of this evidence is from high income countries, highlighting limited research on patient involvement in health care in low and middle income countries (LMICs). Another gap identified from recent reviews is the need to develop effective grievance redressal systems that ensure that patient feedback is responded to[21] and acted upon[13, 22, 23], because patients who do not receive responses to their feedback (especially complaints) are more likely to feel frustrated and disengaged with health services.

Health programmes (including patient feedback systems) are inherently complex, and their success is determined by how the programmes are implemented within the wider health system's context. Theory-driven forms of evaluation help understanding such complexity by studying how different elements of interventions are intertwined[24] and recognising the role of context as a key influence in the production of outcomes[25]. Realist evaluation (RE) is a theory-driven evaluation approach that is increasingly used for studying the implementation of complex interventions within health systems, including in low- and middle-income countries[26, 27]. A realist approach emphasises the contingent nature of programme outcomes and addresses questions about what works, in which setting, for whom, in what circumstances and why[25]. In RE, researchers develop *middle-range theories* that take account of how *Context* (at micro, meso and macro levels) influences intervention processes or *Mechanisms* (e.g. actors' behaviours in implementing intervention) to produce intended and unintended *Outcomes*. This is known as a C-M-O configuration[25], and CMOs allow accounting for all these dimensions, ensuring that all key aspects of the programme are recorded, thus helping to maintain the validity and reliability of results[25].

The Ministry of Health and Family Welfare (MOHFW) in Bangladesh strives to improve the health and wellbeing of vulnerable people (e.g. women and the poor), earmarking >60% of its health spending to the Essential Service Package, a major share of which is spent at the Upazila Health Complex (UHC) level[19]. The UHC is the backbone of Bangladesh's health system as UHCs are the first level referral services from the primary health care facilities (community clinics, union health and family welfare centres). A UHC serves a population of 200,000-400,000 people. Each UHC has a health management committee that monitors service provision, and identifies and addresses emerging problems at UHCs. Membership of UHC management committee include local politicians, health facility managers, civil society representatives and community leaders.

For peer review only - http://bmjopen.bmj.com/site/about/guidelines.xhtml

Since 2009, the MOHFW has implemented a national programme to enhance service users' voice through allowing them to provide feedback on their experiences of using health services in Bangladesh using a Short Messaging Service (SMS) in addition to a more traditional use of suggestion boxes in health facilities[28, 29]. All SMS texts go into a publicly-available national web portal (http://app.dghs.gov.bd/complaintbox/?actn=lstmsg), with many entries containing issues that service users provided feedback on, dates of receipt of feedback and of solution to issues raised by users. This SMS feedback system is monitored by the MOHFW. Each SMS is subsequently followed-up with a phone call to the sender and the local authority of the health facility that the feedback was about. Additional to the SMS feedback and suggestion boxes in each health facility, service users can send feedback directly to UHC management committees at the sub-districts. However, it is unclear, who is responsible for following up issues fed back to grassroots-level health facilities (such as Upazila Health Complexes) and how this follow up is done. It is equally unclear how well the systems of collecting and responding to feedback are integrated with and/or used for supporting staff supervision and performance appraisal and service quality assurance at the Upazila level.

Furthermore, national policymakers recognise that the implementation of the user feedback programme is patchy and needs strengthening. For example, whilst the MOHFW receives approximately 1,000 messages per day, it has only two dedicated staff to follow up SMS feedback. No information is available as to the type of feedback received directly by the health management committees and to what degree the issues are addressed. As a result, ensuring responsive health system in one of the world's most densely populated countries remains a major challenge.

The purpose of this paper is to share the study protocol for a realist evaluation of service user feedback programme in Bangladesh. This paper should be of interest to researchers interested in methodologies for assessing procedures for collecting and addressing service user feedback as well as to policymakers and practitioners interested in designing or evaluating interventions for improving the responsiveness of user feedback systems as part of improving quality of service.

This study aims to better understand the processes of and environment within which service users' feedback is collected, in order to assist policymakers design a comprehensive health systems intervention to make the health system in Bangladesh more responsive to patient feedback. The specific project objectives are to work closely with national and local decision-makers to:

1. Develop an in-depth understanding of the nature, contents of and key reasons why patients provide feedback to health services at district and sub-district (Upazila) levels.
2. Analyse the processes of collecting and responding to service users' feedback at Upazila level, and key contextual facilitators and constraints influencing these processes
3. Assess the approach to and processes of service quality assurance and human resource management, focusing on the use of feedback from service users at Upazila level;
4. Using results of objectives 1-3, to develop a comprehensive and context-specific health systems intervention to improve the use of feedback from service users in quality assurance and human resource management processes at Upazila level.

The achievement of project objectives is a crucial first step of a longer-term plan to implement and assess a comprehensive intervention at larger scale to improve responsiveness of the health system in Bangladesh. The close links between the MOHFW and our project partners in Bangladesh (i.e. ARK Foundation) will facilitate scaling up and developing the national policy to make the country's health system more responsive through better integrating user feedback within quality assurance and Human resource (HR) management.

Study Design and Methods

The 18-month study (January 2017-June 2018) will be implemented in Comilla district that lies southeast of the capital, Dhaka. This district was selected in consultation and discussion with the Ministry of Health and Family Welfare (MOHFW), based on the district receiving frequent user feedbacks, the existence of a motivated district health leadership and our previous successful

For peer review only - http://bmjopen.bmj.com/site/about/guidelines.xhtml

experience of working within the context of Bangladesh. We will purposefully-select two Upazila Health Complexes (UHCs) in Comilla following an initial review of the feedback environment in the district using non-participant observation and review of publicly-available documents. One UHC will have a ‘favourable’ feedback environment (i.e. clear signs explaining where and how to provide feedback and the processes for responding to service user feedback) and the other UHC will have a ‘less favourable’ feedback environment (i.e. no clear signs or processes for dealing with feedback).

Target populations for the study are: a) health service users at UHC, most of whom are groups such as women or poor people, b) service providers and managers, and c) health planners and policymakers. The intervention, to be designed in achieving the objective 4, will include detailed guidance for each target group. For service users, the intervention will detail methods for enhancing their engagement with feedback systems (e.g. improving awareness of strengths of current systems, using health management committees). For providers, managers and policy makers, we will have context-specific tools for improving utilisation of user feedback in quality assurance (e.g. critical incident technique) and HR management (e.g. revised supervision format, contents of staff performance reviews).

Conceptual framework

This is designed as a *multi-disciplinary and mixed-method study* that uses Realist Evaluation to examine user feedback systems in Bangladesh. The realist evaluation approach helps to address questions about what works, for whom, in which circumstances, and why. Researchers use the approach to empirically develop, validate and refine Middle-Range Theories (MRTs) that account for how the *Context* in which interventions are implemented influence intervention *Mechanisms* (e.g. actors’ behaviour in implementing intervention) to produce intended and unintended *Outcomes*. As mentioned earlier, this is known as a C-M-O configuration. Figure 1 below summarises the initial programme theory, which will be continuously validated and refined during data collection and analysis. Detailed C-M-O configurations (e.g. $C_1+M_2=O_2$) will be developed, and will include specific Cs, Ms and Os (identified through objectives 1-3). These, together, will inform the design of the comprehensive health systems intervention in pursuit of the project objective 4.

Initial programme theory: Effective collection of and responses to service user feedback, implemented in the context of effective health management committees at UHC and clear integration between feedback handling and quality assurance and human resource planning and management, should make the Bangladesh’s health system more responsive.

CONTEXTS +	*MECHANISMS* →	*OUTCOMES*
C_1 – Trust, respect and dialogue between service users and health facilities	M_1 - Clear processes for communicating user feedback	O_1 – More responsive health system
C_2 - Effective health management committees	M_2 - Effective feedback review processes and setting agenda for change	O_2 – Improved quality of health services
C_3 – Integration between different components of the health system	M_3 – Continuous monitoring of changes in quality assurance and HR management	O_3 – Improved human resource management processes

Figure 1: Conceptual framework for study

Realist evaluations are method-neutral[25], meaning that researchers can use combinations of methods from different disciplines for the evaluation. In line with this, we will draw upon: process evaluations, statistics, social sciences and health policy and systems research (HPSR), although HPSR can be seen as encompassing all these different disciplines[30]. Process evaluation will be

For peer review only - http://bmjopen.bmj.com/site/about/guidelines.xhtml

used to clarify *causal mechanisms* and *context* of implementation of user feedback systems[31]. Analysis of statistical data from the government web portal and UHC facility records will help clarify the nature of issues reported and their distribution by timing, gender, age or location. Social science methods will guide in-depth analysis of feedback processes, and user and provider experiences of feedback systems. HPSR will help analyse the integration of feedback systems with quality assurance mechanisms and HR management.

Methods

The study will be implemented in the following three phases:

1. Initial theory development: methodology development including developing initial working theories for the patient feedback system (Phase 1)
2. Theory validation and refinement, using continuous rounds of data collection and analysis (Phase 2)
3. Theory consolidation and developing a comprehensive intervention (Phase 3)

Due to the evolving and incremental nature of realist studies, some methodological elements of data collection and analysis will be developed at later stages.

Phase 1: ***Initial theory development*** - which has already started, included three activities and covered the first 2-3 months of the study. First, the research team: i) reviewed publicly-available documents related to the service user feedback systems, ii) visited Upazila Health Complexes to conduct non-participant observations of the complaints environment and iii) held informal discussions with key stakeholders (such as facility-in-charge), to develop and refine programme theories i.e. hypothetical pathways that help explain feedback system(s) and link it/them to existing systems of quality assurance and human resource management within the context Comilla district, Bangladesh. These initial engagements were informal and did not constitute formal interviews requiring ethics clearance (e.g. discussions were not audio-recorded, though stakeholders were made aware of the study using information from relevant participant information sheets). The discussions were organised primarily to facilitate buy-in and project ownership from relevant policymakers and programme implementers. Second, the teams in Leeds and in Bangladesh obtained ethics approvals from the University of Leeds and the Bangladesh Medical Research Council respectively. Third, we developed data collection tools, based on the initial programme theories to be used in the Phase 2 of the study.

At the end of this Phase, the detailed programme theories will be developed to help us explore the relations between context, processes (or mechanisms) and outcomes (also called C-M-O configurations) of the feedback system further in the project.

Phase 2: ***Theory validation and refinement -*** scheduled to start from the second quarter of year-1 and last for about 8-10 months, and will include adaptation of detailed data collection tools, followed by fieldwork to validate and refine programme theories.

We will use different qualitative and quantitative methods to understand and validate programme theories that link context, mechanisms and outcomes of user feedback system. These will be updated, reflecting the evolution of specific MRTs, and will include combinations of:

1. in-depth interviews (IDIs) with service users (about 20 in each UHC, which in our experience is sufficient to capture key perspectives and achieve data saturation) and focus group discussions (FGDs) with community member (2-3 FGDs in each UHC), to explore their knowledge and use of feedback systems. Participants will be purposively selected based on gender, age and use of feedback systems.
2. in-depth interviews with purposefully-selected service providers and managers (about 10 in each UHC, which in our experience is sufficient to capture key perspectives and achieve data saturation) to explore their views on and experience engaging with the user feedback systems.

For peer review only - http://bmjopen.bmj.com/site/about/guidelines.xhtml

3. analysis of country-level secondary data on user feedback from the web portal and UHC records, to understand types of issues, location, gender and age of users who initiated issues.
4. non-participant observation of: feedback environment in the sub-district, health management committee meetings and UHC routine quality assurance and staff management practices.
5. review of key documents e.g. feedback to users and actions taken, meeting minutes, quality assurance guidelines, staff performance appraisal and supervision records.

The respondents for IDIs, identified through purposive sampling will include: UHC managers, health staff, health planners/policymakers at sub-district, district and national levels and selected service users. A detailed list of respondents will be developed after the Phase 1, and snowballing technique will be used to identify any further informants. We will aim to conduct 2-3 focus groups with community members in each UHC, to explore their knowledge and use of feedback systems. However, if we reach data saturation earlier (i.e. when further respondents do not reveal new themes for analysis) these numbers of IDIs and FGDs may decrease in order not to collect any unnecessary data.

The detailed interview and focus group question guides will be developed to inform fieldwork during Phase 2. These will be informed by the study conceptual framework and structured around the study research objectives to explore programme theories developed during the Phase 1. Question guides will be adapted to the different groups of stakeholders, commensurate to their backgrounds, degree of involvement and particular roles in the design and implementation of the patient feedback system. All interviews will be audio-recorded (subject to informed consent), transcribed and where appropriate translated into English for analysis. Framework approach will be used to test hypotheses, while allowing for emergence of new themes and will include stages of familiarisation, coding, indexing and charting, mapping and interpretation[32].

The qualitative and quantitative data collection methods will be integrated throughout, and their combinations will be required to achieve the project objectives. Analysis of qualitative and quantitative data will be done using convergent mixed methods model i.e. involving continuous triangulation of multiple datasets [33], and enable greater understanding of the context of user feedback system, including the links with relevant policies, practices and programmes.

Phase 3: Theory consolidation and developing a comprehensive intervention. During this Phase, we will summarise our refined programme theories in form of middle-range theories, to help articulate a theoretically-robust and empirically-tested model of complex relations between the contexts, mechanisms and outcomes of patient feedback system in Bangladesh.

We will also use this phase to develop a comprehensive health systems intervention to improve utilisation of user feedback in health service quality assurance and human resource management processes at Upazila level. As part of this, we plan to have two workshops with key stakeholders: at the first workshop we will share and discuss results of our analyses and during the second workshop we will facilitate development of a comprehensive health systems intervention by the key health policy actors in Bangladesh. A project work plan is shown in Figure 2

Project Activity \ Year and Quarter	*Year 1*				*Y2*	
	1	*2*	*3*	*4*	*1*	*2*
Initial assessment of feedback environment in the district, selection of UHCs						
Obtaining ethics approvals, finalising and piloting of data collection tools						
Data collection and analysis to continuously validate and refine MRTs						
Workshop 1 with key actors to discuss results of analysis (from objectives 1-3)						
Workshop 2 with key actors to develop intervention (objective 4)						
Continuous engagement with actors, production of materials, research uptake						

Figure 2: Project work plan

For peer review only - http://bmjopen.bmj.com/site/about/guidelines.xhtml

As shown in Figure 2, there are some overlaps among the three project Phases. We do not see the progression between the different phases as a linear process: i.e. as part of the analysis we are likely to identify further hypothetical pathways which may require further data collection and analysis. The specific programme theories that emerge will be continuously refined, and will provide a framework for the data collection and analysis, in line with the principles of the realist evaluation. We will work with decision-makers in a research-policy partnership[34], to facilitate adoption of results into policy.

Ethics and research governance

Ethical approvals for this study were obtained from the School of Medicine Research Ethics Committee at the Faculty of Medicine and Health at the University of Leeds (ref: MREC16-110) and the Bangladesh Medical Research Council (ref: BMRC/NREC/2016-2019/164). These are available in supplementary files 1 and 2 respectively.

The project will be carried out with full respect for current relevant legislations (e.g. the Charter of Fundamental Rights of the EU) and international conventions (e.g. Helsinki Declaration). The methods development, data collection and analysis will take account of the following issues:

- *Anonymity* of study respondents will be preserved where possible and will be ensured at all times if respondent(s) request. Unnecessary collection of personal data will be avoided and respondents will have the right to review study outputs and withdraw consent if necessary. Where personal data is collected, it will be coded, removed from the data for analysis and stored separately from transcripts. Only PIs and designated research personnel in each partner institution will have access to the keys linking the data with the personal information.
- *Informed consent* will be obtained from all study participants and in the case of refusal, alternative means of data collection will be explored (eg. alternative respondents)
- Specific emphasis will be placed on *confidentiality and other data protection issues*, which will include security of data storage and access rights to data. Only members of teams identified by the PIs in each institution will have access to the data. Where project data (eg. interview transcripts) is stored on an institutional server it will be password-protected and only members of the research team will have access to the passwords. The availability of documents on the internet will be with the consent of both project partners.

The project will be implemented according to standard governance practice at the University of Leeds for the implementation of collaborative projects. This includes ensuring: regular communication between the partners and engagement with policymakers and practitioners; quality assurance through regular peer-review both within and between the teams; appropriate mentoring and coaching support of junior researchers; and ensuring equal opportunities to both genders.

Communication and dissemination of results

Adequate communication of results to inform policy and practice is an essential component of any health systems and policy research. We will 'embed' the study into policy and practice, working with national, regional and local actors. This approach, developed by the Nuffield Centre, has been effective in many countries in improving the quality and effectiveness of the implemented programme [35]. Decision-makers at district and MOHFW levels will be continuously engaged throughout the process in a research-policy partnership, to facilitate adoption of effective strategies and tools[34]. The study results will be used – through the 'embedded' research and development approach within policymaking and programme planning – to contribute further improvements in health care provision and achievement of better health outcomes. Specific methods of communicating research findings will include combinations of:

a) Delivering presentations at review meetings at district and national levels in Bangladesh (e.g. semi-annual and annual reviews involving national and international policy actors)
b) Developing newsletters and press-releases aimed at communicating key study findings in ways that are accessible to the general public in Bangladesh and wider within Asia;
c) Developing policy briefs addressed to national and international policymakers and

For peer review only - http://bmjopen.bmj.com/site/about/guidelines.xhtml

practitioners and designed as short and practical documents;
d) Possible interviews in the national media (e.g. radio and television) as well as articles for national newspapers, communicating our findings and educating the public as needed;
e) Developing a dedicated website for the project where the project results will be publicly accessible by national and international decision-makers, practitioners and academics
f) Delivering presentations at national, regional and international conferences and publication of articles in peer-reviewed journals with specific emphasis on open access where feasible
g) Developing a project research report for the funder, with a publishable executive summary.

Building responsive health systems is a priority, both nationally and internationally. The existence of an on-going government programme to enhance users' voice, a clear intention to further strengthen the programme and a strong interest for this research from the national and local decision-makers in Bangladesh, provide an excellent opportunity to generate high-quality evidence and ensure its highest impact on policy and practice in Bangladesh.

Discussion

In this paper, we have reported a study protocol for realist evaluation of patient feedback system in the context of Bangladesh. This is designed as a multi-disciplinary and mixed methods research which aims to better understand the system of patient feedback, in order to assist policymakers design a comprehensive health systems intervention to make the health system in Bangladesh more responsive.

Since the start of the study, three initial hypothetical pathways or initial working theories (IWTs) have been developed at a workshop held in February-March 2017. The first initial working theory (IWT1) focuses of the motivation for and willingness of service users to provide feedback, the second (IWT2) deals with processing and analysis of complaints at Upazila Health Complexes, while the third (IWT3) focuses on acting on user feedback and providing users with relevant feedback for their complaints. These initial working theories (which progressed from the overall programme theory shown in the Figure 1), are currently being further developed using literature review, analysis of key documents and limited number of interviews with key stakeholders as part of the project Phase 1. Each IWT (IWT1, 2 and3) identified specific Cs, Ms and Os developed from researchers' understanding of the user feedback programme, informal engagements with key MOHFW personnel and UHC staff in Bangladesh and review of relevant literature on the subject. The relationships between and amongst these specific Cs, Ms and Os will be explored as part of data collection for the study. The IWTs were subsequently translated into the specific information areas to guide the development of specific tools for primary data collection.

The following aspects of the context within which this study is implemented are worth mentioning. First, the health systems environment in Bangladesh currently promotes evidence-informed health policymaking, as we found within our previous collaborative projects. The commitment by key health decision-makers at Comilla district and the national MOHFW to engage with this study is a particular strength of this project. This will ensure the high ownership and potential for impact of the comprehensive intervention to be developed by the key stakeholders during the latter stages of the project. Second, at theoretical level, the gaps in the literature on user feedback, combined with an increasing interest in applied research focusing on practical issues raised by policymakers and funders, create a favourable environment for the study to generate new knowledge. The study findings will provide a timely contribution to an on-going debate about processes for and effectiveness of user feedback systems in LMICs.

This study has a potential to improve understanding of the functioning of user feedback system in Bangladesh, including in-depth understanding of key contextual factors at macro, meso and micro levels affecting this performance. The study results can be used to achieve improvements in policymaking, health systems strengthening and improvements in health outcomes. In line with this, specific impacts of our study on policy and practice in Bangladesh and internationally include:

For peer review only - http://bmjopen.bmj.com/site/about/guidelines.xhtml

1) Improvements in user feedback systems, implemented to empower the public to hold health system to account and enhance the responsiveness of Bangladesh's health system.
2) Developing local expertise on the design and implementation of context-specific health systems interventions to ensure user feedback is processed and acted upon.
3) Utilisation of innovative, cross-disciplinary, approaches for assessing effectiveness of complex interventions including user feedback systems.
4) Scientific advancement of theories on how to make health systems more responsive in the context of LMICs.

This study will make a vital contribution to health systems responsiveness in Comilla district, and more widely across Bangladesh. Evaluation of complex interventions such as service user feedback systems and their longer-term impact on quality assurance and human resource management requires a comprehensive understanding of intervention context, implementation, mechanisms and outcomes. The multi-disciplinary and mixed-method realist approach that this study adopts will facilitate such evaluation.

For peer review only

For peer review only - http://bmjopen.bmj.com/site/about/guidelines.xhtml

Word count: 4,026 (this excludes, title page, abstract, references, figures and tables)

Declarations

Statement from Corresponding Author

The Corresponding Author has the right to grant on behalf of all authors and does grant on behalf of all authors, an exclusive licence on a worldwide basis to the BMJ Publishing Group Ltd to permit this article (if accepted) to be published in BMJ editions and any other BMJPGL products and sub-licences such use and exploit all subsidiary rights, as set out in our licence.

List of abbreviations

BMRC	Bangladesh medical research council
C-M-O	context-mechanism-outcome
FGD	focus group discussion
HPSR	health policy and systems research
HR	human resource
IDI	in-depth interview
IWT	initial working theory
MOFW	ministry of health and family welfare
MRC	medical research council
MRT	middle-range theory
PHC	primary health care
RE	realist evaluation
SMS	short messaging service
UHC	Upazila Health Complex

Ethics approvals and consent to participate

Ethical approvals for this study were obtained from the School of Medicine Research Ethics Committee at the Faculty of Medicine and Health at the University of Leeds (ref: MREC16-110) and the Bangladesh Medical Research Council (ref: BMRC/NREC/20 16-2019/164). These are available in supplementary files 1 and 2 respectively.

Competing interests

All authors have completed the Unified Competing Interest form and declare: no support from any organisation for the submitted work; no financial relationships with any organisations that might have an interest in the submitted work in the previous three years, no other relationships or activities that could appear to have influenced the submitted work.

Funding

The study protocol reported in this paper has been evaluated through open competitive peer-review process as part of the Joint MRC/ESRC/DFID/Wellcome Trust health systems research initiative call 3, and accepted for funding (grant ref: MR/P004105/1). None of the funders had any role in the design of this study.

Authors' contributions

TM, HE and RH jointly conceived the study; BE, TM, HE, ZA, RH, SN developed the study proposal; BE led the writing of this paper with contributions from TM, HE, RH, ZA, SN. All authors read and approved the final version of the manuscript.

For peer review only

For peer review only - http://bmjopen.bmj.com/site/about/guidelines.xhtml

Word count: 4,026 (this excludes, title page, abstract, references, figures and tables)

Transparency declaration

The lead author affirms that the manuscript is an honest, accurate, and transparent account of the study being reported. No important aspects of the study have been omitted; and any discrepancies from the study as planned have been (or in the case of a study protocol, will be) explained.

Acknowledgements

The study is funded by the Joint MRC/ESRC/Wellcome/DFID Health Systems Research Initiative, Grant reference number MR/P004105/1. All views expressed in this publication are of the authors only. The authors also wish to acknowledge the contributions of staff at the Ministry of Health and Family Welfare of Bangladesh to the study design.

Supplementary file 1 – Ethics approval from the University of Leeds

uploaded as separate file

Supplementary file 2: Ethical approval from the Bangladesh Medical Research Council

uploaded as separate file

References

1. WHO. *Everybody's business-strengthening health systems to improve health outcomes: WHO's framework for action*. Geneva: World Health Organization; 2007.
2. WHO. *Changing Mindsets - Strategy on Health Policy and Systems Research*. Geneva: World Health Organization; 2012.
3. Phillips NM, Street M, Haesler E. A systematic review of reliable and valid tools for the measurement of patient participation in healthcare. *BMJ Quality & Safety* 2016;**25**: 110-117.
4. Health Systems Global. Background to the Vancouver 2016 Symposium theme. In: Health Systems Global, 2015.
5. Lodenstein E, Dieleman M, Gerretsen B, et al. A realist synthesis of the effect of social accountability interventions on health service providers' and policymakers' responsiveness. *Systematic Reviews* 2013;**2**: 98.
6. Molyneux S, Atela M, Angwenyi V, et al. Community accountability at peripheral health facilities: a review of the empirical literature and development of a conceptual framework. *Health Policy and Planning* 2012;**27**: 541-554.
7. Ha BTT, Mirzoev T, Morgan R. Patient complaints in healthcare services in Vietnam's health system. *SAGE Open Medicine* 2015;**3**: 2050312115610127.
8. Thi Thu Ha B, Mirzoev T, Morgan R. Patient complaints in healthcare services in Vietnam's health system. *SAGE Open Medicine* 2015;**3**: 2050312115610127.
9. Reader TW, Gillespie A, Roberts J. Patient complaints in healthcare systems: a systematic review and coding taxonomy. *BMJ Quality & Safety* 2014;**0**: 1-12.
10. Debono D, Travaglia J, University of New South Wales. *Complaints and patient satisfaction: a comprehensive review of the literature*. Syndey, Australia: Centre for Clinical Governance Research, University of New South Wales; 2009.
11. Reader TW, Gillespie A, Roberts J. Patient complaints in healthcare systems: a systematic review and coding taxonomy. *BMJ Quality & Safety* 2014;**0**: 1-12.
12. Hsieh SY. The use of patient complaints to drive quality improvement: an exploratory study in Taiwan. *Health Services Management Research* 2010;**23**: 5-11.
13. Hsieh SY. Healthcare complaints handling systems: a comparison between Britain, Australia and Taiwan. *Health Services Management Research* 2011;**24**: 91-95.

For peer review only - http://bmjopen.bmj.com/site/about/guidelines.xhtml

14. Martineau T, Mirzoev T, Pearson S, et al. Coherence between health policy and human resource strategy: lessons from maternal health in Vietnam, India and China. *Health Policy and Planning* 2015;**30**: 111-120.
15. Mshelia C, Huss R, Mirzoev T, et al. Can action research strengthen district health management and improve health workforce performance? A research protocol. *BMJ Open* 2013;**3**: e003625.
16. Hsieh SY. An exploratory study of complaints handling and nature. *International Journal of Nursing Practice* 2012;**18**: 471-480.
17. Travis P, Bennett S, Haines A, et al. Overcoming health-systems constraints to achieve the Millennium Development Goals. *The Lancet* 2004;**364**: 900-906.
18. WHO. The Millennium Development Goals will not be attained without new research addressing health system constraints to delivering effective interventions: Report of the Task Force on Health Systems Research. In. Geneva, Switzerland: World Health Organization, 2005; 65.
19. Renedo A, Marston CA, Spyridonidis D, et al. Patient and Public Involvement in Healthcare Quality Improvement: How organizations can help patients and professionals to collaborate. *Public Management Review* 2015;**17**: 17-34.
20. Crawford MJ, Rutter D, Manley C, et al. Systematic review of involving patients in the planning and development of health care. *BMJ* 2002;**325**: 1263.
21. O'Mara-Eves A, Brunton G, McDaid D, et al. Community engagement to reduce inequalities in health: a systematic review, meta-analysis and economic analysis. *Public Health Research, No.14* 2013;**1**: i-525.
22. Hsieh SY. A System for Using Patient Complaints as a Trigger to Improve Quality. *Quality Management in Healthcare* 2011;**20**: 343-355.
23. Jonsson PM, Øvretveit J. Patient claims and complaints data for improving patient safety. *International Journal of Health Care Quality Assurance* 2008;**21**: 60-74.
24. Stame N. Theory-Based Evaluation and Types of Complexity. *Evaluation* 2004;**10**: 58-76.
25. Pawson R, Tilley N. *Realistic Evaluation*: London: SAGE Publications; 1997.
26. Maluka S, Kamuzora P, SanSebastián M, et al. Implementing accountability for reasonableness framework at district level in Tanzania: a realist evaluation. *Implementation Science : IS* 2011;**6**: 11-11.
27. Prashanth NS, Marchal B, Hoeree T, et al. How does capacity building of health managers work? A realist evaluation study protocol. *BMJ Open* 2012;**2**: e000882.
28. Naylor M, Huque R, Nash R. *Assessing voice and accountability in health, population and nutrition sector development programme, 2011-2016*. Dhaka, Bangladesh: MOHFW and DFID; 2013.
29. Hutchinson B. Harnessing Technology to Strengthen Accountability: A Collection of Unique Perspectives. In, *IMPACT & INNOVATION SERIES*. Washintong, USA: The National Bureau of Asian Research: Centre for for Health and Aging, 2012.
30. Gilson L. Health Policy and Systems Research. A Methodology Reader. In. Geneva, Switzerland: Alliance for Health Policy and Systems Research, World Health Organization., 2012.
31. MRC. *Developing and Evaluating Complex Interventions*. London: Medical Research Council; 2008.
32. Ritchie J, Spencer L. Qualitative data analysis for applied policy research. *The qualitative researcher's companion* 2002;**573**: 305-329.
33. Ozawa S, Pongpirul K. 10 best resources on … mixed methods research in health systems. *Health Policy and Planning* 2014;**29**: 323-327.
34. Mirzoev TN, Omar MA, Green AT, et al. Research-policy partnerships - experiences of the Mental Health and Poverty Project in Ghana, South Africa, Uganda and Zambia. *Health Research Policy and Systems* 2012;**10**: 30.
35. COMDIS-HSD. *'How To' Guide On Research & Development: The Embedded Approach* Leeds, U.K.: COMDIS-Health Service Delivery; 2012.

For peer review only

For peer review only - http://bmjopen.bmj.com/site/about/guidelines.xhtml

BMJ Open

Protocol for a mixed methods realist evaluation of a health service user feedback system in Bangladesh.

Journal:	*BMJ Open*
Manuscript ID	bmjopen-2017-017743.R1
Article Type:	Protocol
Date Submitted by the Author:	18-May-2017
Complete List of Authors:	Ebenso, Bassey; University of Leeds Faculty of Medicine and Health, Nuffield Centre for International Health and Development<br>Huque, Rumana; ARK FOUNDATION , Road 21, House B130, New DOHS; University of Dhaka Department of Economics, Department of Economics<br>Azdi, Zunayed; ARK FOUNDATION , Road 21, House B130, New DOHS<br>Elsey, Helen; University of Leeds, Nuffield Centre for International Health and Development<br>Nasreen, Shammi; ARK FOUNDATION , Road 21, House B130, New DOHS<br>Mirzoev, Tolib; University of Leeds, Nuffield Centre for International Health and Development
**Primary Subject Heading**:	Patient-centred medicine
Secondary Subject Heading:	Health policy, Health services research, Global health
Keywords:	Realist evaluation, Service User Feedback, Bangladesh, mixed methods, health systems responsiveness

SCHOLARONE™
Manuscripts

only

For peer review only - http://bmjopen.bmj.com/site/about/guidelines.xhtml

Title Page

Protocol for a mixed methods realist evaluation of a health service user feedback system in Bangladesh.

AUTHORS:

- Bassey Ebenso*[1] B.E.Ebenso@leeds.ac.uk
- Rumana Huque[2,3] rumana@arkfoundationbd.org
- Zunayed Azdi[2] azdi@arkfoundationbd.org
- Helen Elsey[1] H.Elsey@leeds.ac.uk
- Shammi Nasreen[2] shammi@arkfoundationbd.org
- Tolib Mirzoev[1] T.mirzoev@leeds.ac.uk

**Corresponding author*

Bassey Ebenso
Nuffield Centre for International Health and Development,
Leeds Institute of Health Sciences
University of Leeds,
Room 10.28, Worsely Building

Clarendon Way,
LS2 9NL
Leeds, UK.

Email: B.E.Ebenso@leeds.ac.uk

Phone: +44 (0)1133439642

Authors institutional addresses:

[1] Nuffield Centre for International Health and Development, University of Leeds, Worsely Building, Clarendon Way, Leeds, UK.
[2] ARK Foundation, Road 21, House B130, New DOHS, Mohakhali, Dhaka, Bangladesh
[3] Department of Economics, University of Dhaka, Dhaka, Bangladesh

KEY WORDS:
Realist evaluation, service user feedback, Bangladesh, mixed methods, health systems responsiveness

For peer review only

For peer review only - http://bmjopen.bmj.com/site/about/guidelines.xhtml

ABSTRACT

Introduction

Responsiveness to service users' views is a widely-recognised objective of health systems. A key component of responsive health systems is effective interaction between users and service providers. Despite a growing literature on patient feedback from high-income settings, less is known about effectiveness of such systems in low and middle income countries.

Methodology and analysis

This paper disseminates the protocol for an 18-month 'RESPOND' project that aims to evaluate the system of collecting and responding to user feedback in Bangladesh. This mixed-method study uses a Realist Evaluation approach to examine user feedback systems at two Upazila Health Complexes in Comilla district of Bangladesh, and comprises three steps: i) initial theory development; ii) theory validation; and iii) theory refinement and development of lessons learned. The project also utilises: i) Process evaluation to understand *causal mechanisms* and *contexts of implementation;* ii) Statistical analysis of patient feedback to clarify the nature of issues reported; iii) Social science methods to illuminate feedback processes and user and provider experiences; and iv) Health policy and systems research to clarify issues related to integration of feedback systems with quality assurance and human resource management. During data analysis, qualitative and quantitative findings will be integrated throughout to help achieve study objectives. Analysis of qualitative and quantitative data will be done using a convergent mixed methods model, involving continuous triangulation of multiple datasets to facilitate greater understanding of the context of user feedback systems including the links with relevant policies, practices and programmes.

Ethics and dissemination

Ethics approvals were obtained from the University of Leeds and the Bangladesh Medical Research Council. All data collected for this study will be anonymised and identifying characteristics of respondents will not appear in a final manuscript or reports. The study findings will be presented at scientific conferences and published in peer-reviewed journals.

KEY WORDS:

Realist evaluation, service user feedback, Bangladesh, mixed methods, health systems responsiveness

For peer review only - http://bmjopen.bmj.com/site/about/guidelines.xhtml

Strengths and limitations of this study

- Adopting a structured realist methodology will shed light on how the *Context* of implementation of user feedback affects intervention *Mechanisms* (e.g. actors' behaviour in implementing systems) to produce intended and unintended *Outcomes.*

- The gaps in literature on user feedback combined with a focus on practical issues raised by policymakers and funders, create a favourable environment for the study to generate new knowledge

- Involving health managers and decision-makers in designing and assessing performance of user feedback systems will foster ownership of the results and ensure sustainability of interventions to improve health systems responsiveness in Bangladesh.

- Managing various disciplines included in the study design (i.e. process evaluation, statistical analysis, social science methods, and health policy and systems research) can be a challenge.

- Implementing the study in only one district, may affect generalizability of study findings to the whole country.

For peer review only

For peer review only - http://bmjopen.bmj.com/site/about/guidelines.xhtml

Introduction

Responsiveness to service user views is a widely-recognised objective of national health systems[1-3]. Responsive health systems anticipate and adapt to future health needs, and harness emerging opportunities to promote universal access to effective interventions[4]. A key component of responsive health systems is effective interaction between service users and service providers or managers[5-8]. This interaction is important in two ways. First, it gives service users the opportunity to provide feedback on issues such as their experiences of the care they received, perception of staff expertise, availability of supplies etc.[8-11]. We use the term 'feedback' as this includes both complaints (i.e. grievances) and praises (i.e. positive reflections), from service users. Second, the interaction provides the health system with the opportunity to collect, respond to, and utilise, user feedback in e.g. improving health service quality[12, 13] or strengthening human resource (HR) management processes[13-15]. There are two approaches to collecting user feedback: one in which data collection is led by service providers, researchers or managers (e.g. using surveys, critical incident techniques, case studies and interviews)[10] and the other, in which service users actively provide information (e.g. through suggestions boxes and telephone hotlines)[16]. The RESPOND project focuses on user-initiated feedback.[17, 18]

Health service users, or patients, across the world are increasingly asked to voice their opinions about service development and/or provide feedback on their experiences of health care services[19]. A systematic review of the impact of involving users in healthcare found evidence of improved health services[20]. However, much of this evidence is from high income countries, highlighting limited research on patient involvement in health care in low and middle income countries (LMICs). Another gap identified from recent reviews is the need to develop effective grievance redressal systems that ensure that patient feedback is responded to[21] and acted upon[13, 22, 23], because patients who do not receive responses to their feedback (especially complaints) are more likely to feel frustrated and disengaged with health services.

Health programmes (including patient feedback systems) are inherently complex, and their success is determined by how the programmes are implemented within the wider health system's context. Theory-driven forms of evaluation help understanding such complexity by studying how different elements of interventions are intertwined[24] and recognising the role of context as a key influence in the production of outcomes[25]. Realist evaluation (RE) is a theory-driven evaluation approach that is increasingly used for studying the implementation of complex interventions within health systems, including in low- and middle-income countries[26, 27]. A realist approach emphasises the contingent nature of programme outcomes and addresses questions about what works, in which setting, for whom, in what circumstances and why[25]. In RE, researchers develop *middle-range theories* that take account of how *Context* (at micro, meso and macro levels) influences intervention processes or *Mechanisms* (e.g. actors' behaviours in implementing intervention) to produce intended and unintended *Outcomes*. This is known as a C-M-O configuration[25], and CMOs allow accounting for all these dimensions, ensuring that all key aspects of the programme are recorded, thus helping to maintain the validity and reliability of results[25].

The Ministry of Health and Family Welfare (MOHFW) in Bangladesh strives to improve the health and wellbeing of vulnerable people (e.g. women and the poor), earmarking >60% of its health spending to the Essential Service Package, a major share of which is spent at the Upazila Health Complex (UHC) level[19]. The UHC is the backbone of Bangladesh's health system as UHCs are the first level referral services from the primary health care facilities (community clinics, union health and family welfare centres). A UHC serves a population of 200,000-400,000 people. Each UHC has a health management committee that monitors service provision, and identifies and addresses emerging problems at UHCs. Membership of UHC management committee include local politicians, health facility managers, civil society representatives and community leaders.

Since 2009, the MOHFW has implemented a national programme to enhance service users' voice through allowing them to provide feedback on their experiences of using health services in Bangladesh using a Short Messaging Service (SMS) in addition to a more traditional use of suggestion boxes in health facilities[28, 29]. All SMS texts go into a publicly-available national web

For peer review only - http://bmjopen.bmj.com/site/about/guidelines.xhtml

portal (http://app.dghs.gov.bd/complaintbox/?actn=lstmsg), with many entries containing issues that service users provided feedback on, dates of receipt of feedback and of solution to issues raised by users. This SMS feedback system is monitored by the MOHFW. Each SMS is subsequently followed-up with a phone call to the sender and the local authority of the health facility that the feedback was about. Additional to the SMS feedback and suggestion boxes in each health facility, service users can send feedback directly to UHC management committees at the sub-districts. However, it is unclear, who is responsible for following up issues fed back to grassroots-level health facilities (such as Upazila Health Complexes) and how this follow up is done. It is equally unclear how well the systems of collecting and responding to feedback are integrated with and/or used for supporting staff supervision and performance appraisal and service quality assurance at the Upazila level.

Furthermore, national policymakers recognise that the implementation of the user feedback programme is patchy and needs strengthening. For example, whilst the MOHFW receives approximately 1,000 messages per day, it has only two dedicated staff to follow up SMS feedback. No information is available as to the type of feedback received directly by the health management committees and to what degree the issues are addressed. As a result, ensuring responsive health system in one of the world's most densely populated countries remains a major challenge.

The purpose of this paper is to share the study protocol for a realist evaluation of service user feedback programme in Bangladesh. This paper should be of interest to researchers interested in methodologies for assessing procedures for collecting and addressing service user feedback as well as to policymakers and practitioners interested in designing or evaluating interventions for improving the responsiveness of user feedback systems as part of improving quality of service.

This study aims to better understand the processes of and environment within which service users' feedback is collected, in order to assist policymakers design a comprehensive health systems intervention to make the health system in Bangladesh more responsive to patient feedback. The specific project objectives are to work closely with national and local decision-makers to:

1. Develop an in-depth understanding of the nature, contents of and key reasons why patients provide feedback to health services at district and sub-district (Upazila) levels.
2. Analyse the processes of collecting and responding to service users' feedback at Upazila level, and key contextual facilitators and constraints influencing these processes
3. Assess the approach to and processes of service quality assurance and human resource management, focusing on the use of feedback from service users at Upazila level;
4. Using results of objectives 1-3, to develop a comprehensive and context-specific health systems intervention to improve the use of feedback from service users in quality assurance and human resource management processes at Upazila level.

The achievement of project objectives is a crucial first step of a longer-term plan to implement and assess a comprehensive intervention at larger scale to improve responsiveness of the health system in Bangladesh. The close links between the MOHFW and our project partners in Bangladesh (i.e. ARK Foundation) will facilitate scaling up and developing the national policy to make the country's health system more responsive through better integrating user feedback within quality assurance and Human resource (HR) management.

There are no widely used systems for disseminating realist evaluation protocols. Therefore in reporting our study protocol, we draw on different checklists for reporting empirical results. These include: COREQ standards for reporting qualitative research[30] and a recently published RAMESES II reporting standards for realist evaluations[31, 32]. In this protocol, we outline the study design and methods including study setting, conceptual framework, data collection and analysis methods. We also explain researchers' background, key ethics and research governance issues, and our approach to dissemination.

For peer review only - http://bmjopen.bmj.com/site/about/guidelines.xhtml

Study Design and Methods

Study setting and target population

The 18-month study (January 2017-June 2018) will be implemented in Comilla district that lies southeast of the capital, Dhaka. This district was selected in consultation and discussion with the Ministry of Health and Family Welfare (MOHFW), based on the district receiving frequent user feedbacks, the existence of a motivated district health leadership and our previous successful experience of working within the context of Bangladesh. We will purposefully-select two Upazila Health Complexes (UHCs) in Comilla following an initial review of the feedback environment in the district using non-participant observation and review of publicly-available documents. One UHC will have a 'favourable' feedback environment (i.e. clear signs explaining where and how to provide feedback and the processes for responding to service user feedback) and the other UHC will have a 'less favourable' feedback environment (i.e. no clear signs or processes for dealing with feedback).

Target populations for the study are: a) health service users at UHC, most of whom are groups such as women or poor people, b) service providers and managers, and c) health planners and policymakers. The intervention, to be designed in achieving the objective 4, will include detailed guidance for each target group. For service users, the intervention will detail methods for enhancing their engagement with feedback systems (e.g. improving awareness of strengths of current systems, using health management committees). For providers, managers and policy makers, we will have context-specific tools for improving utilisation of user feedback in quality assurance (e.g. critical incident technique) and HR management (e.g. revised supervision format, contents of staff performance reviews).

Conceptual framework

This is designed as a *multi-disciplinary and mixed-method study* that uses Realist Evaluation to examine user feedback systems in Bangladesh. The realist evaluation approach helps to address questions about what works, for whom, in which circumstances, and why. Researchers use the approach to empirically develop, validate and refine Middle-Range Theories (MRTs) that account for how the *Context* in which interventions are implemented influence intervention *Mechanisms* (e.g. actors' behaviour in implementing intervention) to produce intended and unintended *Outcomes*. As mentioned earlier, this is known as a C-M-O configuration. Figure 1 below summarises the initial programme theory, which will be continuously validated and refined during data collection and analysis. Detailed C-M-O configurations (e.g. $C_1+M_2=O_2$) will be developed, and will include specific Cs, Ms and Os (identified through objectives 1-3). These, together, will inform the design of the comprehensive health systems intervention in pursuit of the project objective 4.

INSERT FIGURE 1 HERE

Realist evaluations are method-neutral[25], meaning that researchers can use combinations of methods from different disciplines for the evaluation[33]. In line with this, we will draw upon: process evaluations, statistics, social sciences and health policy and systems research (HPSR), although HPSR can be seen as encompassing all these different disciplines[34]. Process evaluation will be used to clarify *causal mechanisms* and *context* of implementation of user feedback systems[35]. Analysis of statistical data from the government web portal and UHC facility records will help clarify the nature of issues reported and their distribution by timing, gender, age or location. Social science methods will guide in-depth analysis of feedback processes, and user and provider experiences of feedback systems. HPSR will help analyse the integration of feedback systems with quality assurance mechanisms and HR management.

Methods of data collection and sampling

The study will be implemented in the following three phases:

For peer review only - http://bmjopen.bmj.com/site/about/guidelines.xhtml

1. Initial theory development: methodology development including developing initial working theories for the patient feedback system (Phase 1)
2. Theory validation and refinement, using continuous rounds of data collection and analysis (Phase 2)
3. Theory consolidation and developing a comprehensive intervention (Phase 3)

Due to the evolving and incremental nature of realist studies, some methodological elements of data collection and analysis will be developed at later stages.

Phase 1: ***Initial theory development*** - which has already started, included three activities and covered the first 2-3 months of the study. First, the research team: i) reviewed publicly-available documents related to the service user feedback systems, ii) visited Upazila Health Complexes to conduct non-participant observations of the complaints environment and iii) held informal discussions with 3-5 key stakeholders (such as facility-in-charge and policymakers), to develop and refine programme theories i.e. hypothetical pathways that help explain feedback system(s) and link it/them to existing systems of quality assurance and human resource management within the context Comilla district, Bangladesh. These initial engagements were informal and did not constitute formal interviews requiring ethics clearance (e.g. discussions were not audio-recorded, though stakeholders were made aware of the study using information from relevant participant information sheets). The discussions were organised primarily to facilitate buy-in and project ownership from relevant policymakers and programme implementers. Second, the teams in Leeds and in Bangladesh obtained ethics approvals from the University of Leeds and the Bangladesh Medical Research Council respectively. Third, we will develope data collection tools, based on the initial programme theories to be used in the Phase 2 of the study.

At the end of this Phase, the detailed programme theories will be developed to help us explore the relations between context, processes (or mechanisms) and outcomes (also called C-M-O configurations) of the feedback system further in the project.

Phase 2: ***Theory validation and refinement -*** scheduled to start from the second quarter of year-1 and last for about 8-10 months, and will include adaptation and pilot testing of generic qualitative and quantitative data collection tools, followed by the bulk of fieldwork to validate and refine programme theories.

We will use different qualitative and quantitative methods to understand and validate programme theories that link context, mechanisms and outcomes of user feedback system. These will be updated, reflecting the evolution of specific MRTs, and will include combinations of:

1. in-depth interviews (IDIs) with service users (about 20 in each UHC, which in our experience is sufficient to capture key perspectives and achieve data saturation) and focus group discussions (FGDs) with community member (2-3 FGDs in each UHC), to explore their knowledge and use of feedback systems. Participants will be purposively selected based on gender, age and use of feedback systems.
2. in-depth interviews with purposefully-selected service providers and managers (about 10 in each UHC, which in our experience is sufficient to capture key perspectives and achieve data saturation) to explore their views on and experience engaging with the user feedback systems.
3. analysis of country-level secondary data on user feedback from the web portal and UHC records, to understand types of issues, location, gender and age of users who initiated issues.
4. non-participant observation of: feedback environment in the sub-district, health management committee meetings and UHC routine quality assurance and staff management practices.
5. review of key documents e.g. feedback to users and actions taken, meeting minutes, quality assurance guidelines, staff performance appraisal and supervision records.

The respondents for IDIs, identified through purposive sampling will include: UHC managers, health staff, health planners/policymakers at sub-district, district and national levels and selected service users. A detailed list of respondents will be developed after the Phase 1, and snowballing technique will be used to identify any further informants. We will aim to conduct 2-3 focus groups

For peer review only - http://bmjopen.bmj.com/site/about/guidelines.xhtml

with community members in each UHC, to explore their knowledge and use of feedback systems. However, if we reach data saturation earlier (i.e. when further respondents do not reveal new themes for analysis) these numbers of IDIs and FGDs may decrease in order not to collect any unnecessary data. Different experiences of the user feedback system are expected to emerge within different sub-groups of respondents. So both men and women and all age groups in the above sub-groups are considered for inclusion (18–65 years).

The detailed interview and focus group question guides will be developed to inform fieldwork during Phase 2. These will be informed by the study conceptual framework and structured around the study research objectives to explore programme theories developed during the Phase 1. Question guides will be adapted to the different groups of stakeholders, commensurate to their backgrounds, degree of involvement and particular roles in the design and implementation of the patient feedback system.

Data analysis

All interviews will be audio-recorded (subject to informed consent), transcribed and where appropriate translated into English for analysis. Framework approach will be used to test hypotheses, while allowing for emergence of new themes and will include stages of familiarisation, coding, indexing and charting, mapping and interpretation[36]. The qualitative and quantitative data collection methods will be integrated throughout, and their combinations will be required to achieve the project objectives. Analysis of qualitative and quantitative data will be done using convergent mixed methods model i.e. involving continuous triangulation of multiple datasets [37], and enable greater understanding of the context of user feedback system, including the links with relevant policies, practices and programmes. We will share interpretations and summary findings of our analysis with key stakeholders. This is discussed below as part of the first workshop of phase 3.

Phase 3: Theory consolidation and developing a comprehensive intervention. During this Phase, we will summarise our refined programme theories in form of middle-range theories, to help articulate a theoretically-robust and empirically-tested model of complex relations between the contexts, mechanisms and outcomes of patient feedback system in Bangladesh.

We will also use this phase to develop a comprehensive health systems intervention to improve utilisation of user feedback in health service quality assurance and human resource management processes at Upazila level. As part of this, we plan to have two workshops with key stakeholders. The first workshop will be *used to share and discuss results of our analyses with key stakeholders* for comment and correction, to ensure our interpretations and conclusions match participants beliefs and experiences of user feedback system. During the second workshop, we will facilitate *development of a comprehensive health systems intervention by the key health policy actors* in Bangladesh. A project work plan is shown in Figure 2.

INSERT FIGURE 2 HERE

As shown in Figure 2, there are some overlaps among the three project Phases. We do not see the progression between the different phases as a linear process: i.e. as part of the analysis we are likely to identify further hypothetical pathways which may require further data collection and analysis. The specific programme theories that emerge will be continuously refined, and will provide a framework for the data collection and analysis, in line with the principles of the realist evaluation. We will work with decision-makers in a research-policy partnership[38], to facilitate adoption of results into policy.

For peer review only - http://bmjopen.bmj.com/site/about/guidelines.xhtml

Researchers' background

The research team comprises 3 males (BE, TM, ZA) and 3 females (HE, RH, SN). BE is a research fellow in health policy and systems research at the university of Leeds, UK and has expertise in methodology development for mixed-methods evaluation of complex applied healthcare interventions. RH is a professor of Health Economics at the University of Dhaka, Bangladesh. She has a background in health systems strengthening. TM is an associate professors of health policy and systems research while HE is associate professor of Public Health at the University of Leeds and they are both experienced in applying realist evaluation methodologies. ZA and SN both hold Masters of Public Health and serve as research staff at the ARK Foundation in Dhaka, Bangladesh. They apply qualitative and realist evaluation methods to studies of maternal and child health in Bangladesh.

The qualitative interviews will be conducted by ZA and SN, who are trained and experienced in conducting and analysing data from patients, providers and policymakers in the context of healthcare provision in Bangladesh. They have no direct connections to the participants or study sites in Comilla district as their qualitative interview training and experiences (including in realist evaluation interviews) were acquired in other districts of Bangladesh, so their distance from participants and studying the sites should constitute a strength. However, as Stanley and Nayar[39] recommend a reflexive approach to managing the researcher-participant relationships, ZA and SN will maintain a research journal that captures their experiences of researcher-participant relationship with study respondents', as a way to ensue study rigour. The journal will explain the reasoning behind decisions made during data analysis and include the pros and cons of a distant relationship with research participants on eliciting research data that is a true reflection of participants' beliefs.

Ethics and research governance

Ethical approvals for this study were obtained from the School of Medicine Research Ethics Committee at the Faculty of Medicine and Health at the University of Leeds (ref: MREC16-110) and the Bangladesh Medical Research Council (ref: BMRC/NREC/2016-2019/164). These are available in supplementary files.

The project will be carried out with full respect for current relevant legislations (e.g. the Charter of Fundamental Rights of the EU) and international conventions (e.g. Helsinki Declaration). The methods development, data collection and analysis will take account of the following issues:

- *Anonymity* of study respondents will be preserved where possible and will be ensured at all times if respondent(s) request. Unnecessary collection of personal data will be avoided and respondents will have the right to review study outputs and withdraw consent if necessary. Where personal data is collected, it will be coded, removed from the data for analysis and stored separately from transcripts. Only PIs and designated research personnel in each partner institution will have access to the keys linking the data with the personal information.
- *Informed consent* will be obtained from all study participants and in the case of refusal, alternative means of data collection will be explored (eg. alternative respondents)
- Specific emphasis will be placed on *confidentiality and other data protection issues*, which will include security of data storage and access rights to data. Only members of teams identified by the PIs in each institution will have access to the data. Where project data (eg. interview transcripts) is stored on an institutional server it will be password-protected and only members of the research team will have access to the passwords. The availability of documents on the internet will be with the consent of both project partners.

The project will be implemented according to standard governance practice at the University of Leeds for the implementation of collaborative projects. This includes ensuring: regular communication between the partners and engagement with policymakers and practitioners; quality assurance through regular peer-review both within and between the teams; appropriate mentoring and coaching support of junior researchers; and ensuring equal opportunities to both genders.

For peer review only - http://bmjopen.bmj.com/site/about/guidelines.xhtml

Communication and dissemination of results

Adequate communication of results to inform policy and practice is an essential component of any health systems and policy research. We will 'embed' the study into policy and practice, working with national, regional and local actors. This approach, developed by the Nuffield Centre, has been effective in many countries in improving the quality and effectiveness of the implemented programme [40]. Decision-makers at district and MOHFW levels will be continuously engaged throughout the process in a research-policy partnership, to facilitate adoption of effective strategies and tools[38]. The study results will be used – through the 'embedded' research and development approach within policymaking and programme planning – to contribute further improvements in health care provision and achievement of better health outcomes. Specific methods of communicating research findings will include combinations of:

a) Delivering presentations at review meetings at district and national levels in Bangladesh (e.g. semi-annual and annual reviews involving national and international policy actors)
b) Developing newsletters and press-releases aimed at communicating key study findings in ways that are accessible to the general public in Bangladesh and wider within Asia;
c) Developing policy briefs addressed to national and international policymakers and practitioners and designed as short and practical documents;
d) Possible interviews in the national media (e.g. radio and television) as well as articles for national newspapers, communicating our findings and educating the public as needed;
e) Developing a dedicated website for the project where the project results will be publicly accessible by national and international decision-makers, practitioners and academics
f) Delivering presentations at national, regional and international conferences and publication of articles in peer-reviewed journals with specific emphasis on open access where feasible
g) Developing a project research report for the funder, with a publishable executive summary.

Building responsive health systems is a priority, both nationally and internationally. The existence of an on-going government programme to enhance users' voice, a clear intention to further strengthen the programme and a strong interest for this research from the national and local decision-makers in Bangladesh, provide an excellent opportunity to generate high-quality evidence and ensure its highest impact on policy and practice in Bangladesh.

Discussion

In this paper, we have reported a study protocol for realist evaluation of patient feedback system in the context of Bangladesh. This is designed as a multi-disciplinary and mixed methods research which aims to better understand the system of patient feedback, in order to assist policymakers design a comprehensive health systems intervention to make the health system in Bangladesh more responsive.

Since the start of the study, three initial hypothetical pathways or initial working theories (IWTs) have been developed at a workshop held in February-March 2017. The first initial working theory (IWT1) focuses of the motivation for and willingness of service users to provide feedback, the second (IWT2) deals with processing and analysis of complaints at Upazila Health Complexes, while the third (IWT3) focuses on acting on user feedback and providing users with relevant feedback for their complaints. These initial working theories (which progressed from the overall programme theory shown in the Figure 1), are currently being further developed using literature review, analysis of key documents and limited number of interviews with key stakeholders as part of the project Phase 1. Each IWT (IWT1, 2 and3) identified specific Cs, Ms and Os developed from researchers' understanding of the user feedback programme, informal engagements with key MOHFW personnel and UHC staff in Bangladesh and review of relevant literature on the subject. The relationships between and amongst these specific Cs, Ms and Os will be explored as part of data collection for the study. The IWTs were subsequently translated into the specific information areas to guide the development of specific tools for primary data collection.

The following aspects of the context within which this study is implemented are worth mentioning. First, the health systems environment in Bangladesh currently promotes evidence-informed health

For peer review only - http://bmjopen.bmj.com/site/about/guidelines.xhtml

policymaking, as we found within our previous collaborative projects. The commitment by key health decision-makers at Comilla district and the national MOHFW to engage with this study is a particular strength of this project. This will ensure the high ownership and potential for impact of the comprehensive intervention to be developed by the key stakeholders during the latter stages of the project. Second, at theoretical level, the gaps in the literature on user feedback, combined with an increasing interest in applied research focusing on practical issues raised by policymakers and funders, create a favourable environment for the study to generate new knowledge. The study findings will provide a timely contribution to an on-going debate about processes for and effectiveness of user feedback systems in LMICs.

This study has a potential to improve understanding of the functioning of user feedback system in Bangladesh, including in-depth understanding of key contextual factors at macro, meso and micro levels affecting this performance. The study results can be used to achieve improvements in policymaking, health systems strengthening and improvements in health outcomes. In line with this, specific impacts of our study on policy and practice in Bangladesh and internationally include:

1) Improvements in user feedback systems, implemented to empower the public to hold health system to account and enhance the responsiveness of Bangladesh's health system.
2) Developing local expertise on the design and implementation of context-specific health systems interventions to ensure user feedback is processed and acted upon.
3) Utilisation of innovative, cross-disciplinary, approaches for assessing effectiveness of complex interventions including user feedback systems.
4) Scientific advancement of theories on how to make health systems more responsive in the context of LMICs.

This study will make a vital contribution to health systems responsiveness in Comilla district, and more widely across Bangladesh. Evaluation of complex interventions such as service user feedback systems and their longer-term impact on quality assurance and human resource management requires a comprehensive understanding of intervention context, implementation, mechanisms and outcomes. The multi-disciplinary and mixed-method realist approach that this study adopts will facilitate such evaluation.

For peer review only - http://bmjopen.bmj.com/site/about/guidelines.xhtml

Declarations

Statement from Corresponding Author

The Corresponding Author has the right to grant on behalf of all authors and does grant on behalf of all authors, an exclusive licence on a worldwide basis to the BMJ Publishing Group Ltd to permit this article (if accepted) to be published in BMJ editions and any other BMJPGL products and sub-licences such use and exploit all subsidiary rights, as set out in our licence.

List of abbreviations

BMRC	Bangladesh medical research council
C-M-O	context-mechanism-outcome
FGD	focus group discussion
HPSR	health policy and systems research
HR	human resource
IDI	in-depth interview
IWT	initial working theory
MOFW	ministry of health and family welfare
MRC	medical research council
MRT	middle-range theory
PHC	primary health care
RE	realist evaluation
SMS	short messaging service
UHC	Upazila Health Complex

Ethics approvals and consent to participate

Ethical approvals for this study were obtained from the School of Medicine Research Ethics Committee at the Faculty of Medicine and Health at the University of Leeds (ref: MREC16-110) and the Bangladesh Medical Research Council (ref: BMRC/NREC/20 16-2019/164).

Competing interests

All authors have completed the Unified Competing Interest form and declare: no support from any organisation for the submitted work; no financial relationships with any organisations that might have an interest in the submitted work in the previous three years, no other relationships or activities that could appear to have influenced the submitted work.

Funding

The study protocol reported in this paper has been evaluated through open competitive peer-review process as part of the Joint MRC/ESRC/DFID/Wellcome Trust health systems research initiative call 3, and accepted for funding (grant ref: MR/P004105/1). None of the funders had any role in the design of this study.

Authors' contributions

TM, HE and RH jointly conceived the study; BE, TM, HE, ZA, RH, SN developed the study proposal; BE led the writing of this paper with contributions from TM, HE, RH, ZA, SN. All authors read and approved the final version of the manuscript.

For peer review only - http://bmjopen.bmj.com/site/about/guidelines.xhtml

Transparency declaration

The lead author affirms that the manuscript is an honest, accurate, and transparent account of the study being reported. No important aspects of the study have been omitted; and any discrepancies from the study as planned have been (or in the case of a study protocol, will be) explained.

Acknowledgements

The study is funded by the Joint MRC/ESRC/Wellcome/DFID Health Systems Research Initiative, Grant reference number MR/P004105/1. All views expressed in this publication are of the authors only. The authors also wish to acknowledge the contributions of staff at the Ministry of Health and Family Welfare of Bangladesh to the study design.

References

1. WHO. *Everybody's business-strengthening health systems to improve health outcomes: WHO's framework for action*. Geneva: World Health Organization; 2007.
2. WHO. *Changing Mindsets - Strategy on Health Policy and Systems Research*. Geneva: World Health Organization; 2012.
3. Phillips NM, Street M, Haesler E. A systematic review of reliable and valid tools for the measurement of patient participation in healthcare. *BMJ Quality & Safety* 2016;**25**: 110-117.
4. Health Systems Global. Background to the Vancouver 2016 Symposium theme. In: Health Systems Global, 2015.
5. Lodenstein E, Dieleman M, Gerretsen B, et al. A realist synthesis of the effect of social accountability interventions on health service providers' and policymakers' responsiveness. *Systematic Reviews* 2013;**2**: 98.
6. Molyneux S, Atela M, Angwenyi V, et al. Community accountability at peripheral health facilities: a review of the empirical literature and development of a conceptual framework. *Health Policy and Planning* 2012;**27**: 541-554.
7. Ha BTT, Mirzoev T, Morgan R. Patient complaints in healthcare services in Vietnam's health system. *SAGE Open Medicine* 2015;**3**: 2050312115610127.
8. Thi Thu Ha B, Mirzoev T, Morgan R. Patient complaints in healthcare services in Vietnam's health system. *SAGE Open Medicine* 2015;**3**: 2050312115610127.
9. Reader TW, Gillespie A, Roberts J. Patient complaints in healthcare systems: a systematic review and coding taxonomy. *BMJ Quality & Safety* 2014;**0**: 1-12.
10. Debono D, Travaglia J, University of New South Wales. *Complaints and patient satisfaction: a comprehensive review of the literature*. Syndey, Australia: Centre for Clinical Governance Research, University of New South Wales; 2009.
11. Reader TW, Gillespie A, Roberts J. Patient complaints in healthcare systems: a systematic review and coding taxonomy. *BMJ Quality & Safety* 2014;**0**: 1-12.
12. Hsieh SY. The use of patient complaints to drive quality improvement: an exploratory study in Taiwan. *Health Services Management Research* 2010;**23**: 5-11.
13. Hsieh SY. Healthcare complaints handling systems: a comparison between Britain, Australia and Taiwan. *Health Services Management Research* 2011;**24**: 91-95.
14. Martineau T, Mirzoev T, Pearson S, et al. Coherence between health policy and human resource strategy: lessons from maternal health in Vietnam, India and China. *Health Policy and Planning* 2015;**30**: 111-120.
15. Mshelia C, Huss R, Mirzoev T, et al. Can action research strengthen district health management and improve health workforce performance? A research protocol. *BMJ Open* 2013;**3**: e003625.
16. Hsieh SY. An exploratory study of complaints handling and nature. *International Journal of Nursing Practice* 2012;**18**: 471-480.
17. Travis P, Bennett S, Haines A, et al. Overcoming health-systems constraints to achieve the Millennium Development Goals. *The Lancet* 2004;**364**: 900-906.

For peer review only - http://bmjopen.bmj.com/site/about/guidelines.xhtml

For peer review only

18. WHO. The Millennium Development Goals will not be attained without new research addressing health system constraints to delivering effective interventions: Report of the Task Force on Health Systems Research. In. Geneva, Switzerland: World Health Organization, 2005; 65.
19. Renedo A, Marston CA, Spyridonidis D, et al. Patient and Public Involvement in Healthcare Quality Improvement: How organizations can help patients and professionals to collaborate. *Public Management Review* 2015;**17**: 17-34.
20. Crawford MJ, Rutter D, Manley C, et al. Systematic review of involving patients in the planning and development of health care. *BMJ* 2002;**325**: 1263.
21. O'Mara-Eves A, Brunton G, McDaid D, et al. Community engagement to reduce inequalities in health: a systematic review, meta-analysis and economic analysis. *Public Health Research, No.14* 2013;**1**: i-525.
22. Hsieh SY. A System for Using Patient Complaints as a Trigger to Improve Quality. *Quality Management in Healthcare* 2011;**20**: 343-355.
23. Jonsson PM, Øvretveit J. Patient claims and complaints data for improving patient safety. *International Journal of Health Care Quality Assurance* 2008;**21**: 60-74.
24. Stame N. Theory-Based Evaluation and Types of Complexity. *Evaluation* 2004;**10**: 58-76.
25. Pawson R, Tilley N. *Realistic Evaluation*: London: SAGE Publications; 1997.
26. Maluka S, Kamuzora P, SanSebastián M, et al. Implementing accountability for reasonableness framework at district level in Tanzania: a realist evaluation. *Implementation Science : IS* 2011;**6**: 11-11.
27. Prashanth NS, Marchal B, Hoeree T, et al. How does capacity building of health managers work? A realist evaluation study protocol. *BMJ Open* 2012;**2**: e000882.
28. Naylor M, Huque R, Nash R. *Assessing voice and accountability in health, population and nutrition sector development programme, 2011-2016*. Dhaka, Bangladesh: MOHFW and DFID; 2013.
29. Hutchinson B. Harnessing Technology to Strengthen Accountability: A Collection of Unique Perspectives. In, *IMPACT & INNOVATION SERIES*. Washintong, USA: The National Bureau of Asian Research: Centre for for Health and Aging, 2012.
30. Tong A, Sainsbury P, Craig J. Consolidated criteria for reporting qualitative research (COREQ): a 32-item checklist for interviews and focus groups. *International Journal for Quality in Health Care* 2007;**19**: 349-357.
31. Greenhalgh T, Wong G, Jagosh J, et al. Protocol—the RAMESES II study: developing guidance and reporting standards for realist evaluation. *BMJ Open* 2015;**5**.
32. Wong G, Westhorp G, Manzano A, et al. RAMESES II reporting standards for realist evaluations. *BMC Medicine* 2016;**14**: 96.
33. Mirzoev T, Etiaba E, Ebenso B, et al. Study protocol: realist evaluation of effectiveness and sustainability of a community health workers programme in improving maternal and child health in Nigeria. *Implementation Science* 2016;**11**: 1-11.
34. Gilson L. Health Policy and Systems Research. A Methodology Reader. In. Geneva, Switzerland: Alliance for Health Policy and Systems Research, World Health Organization., 2012.
35. MRC. *Developing and Evaluating Complex Interventions*. London: Medical Research Council; 2008.
36. Ritchie J, Spencer L. Qualitative data analysis for applied policy research. *The qualitative researcher's companion* 2002;**573**: 305-329.
37. Ozawa S, Pongpirul K. 10 best resources on … mixed methods research in health systems. *Health Policy and Planning* 2014;**29**: 323-327.
38. Mirzoev TN, Omar MA, Green AT, et al. Research-policy partnerships - experiences of the Mental Health and Poverty Project in Ghana, South Africa, Uganda and Zambia. *Health Research Policy and Systems* 2012;**10**: 30.
39. Stanley M, Nayar S. 2 Deepening understandings. *Qualitative Research Methodologies for Occupational Science and therapy* 2014: 8.
40. COMDIS-HSD. *'How To' Guide On Research & Development: The Embedded Approach* Leeds, U.K.: COMDIS-Health Service Delivery; 2012.

For peer review only - http://bmjopen.bmj.com/site/about/guidelines.xhtml

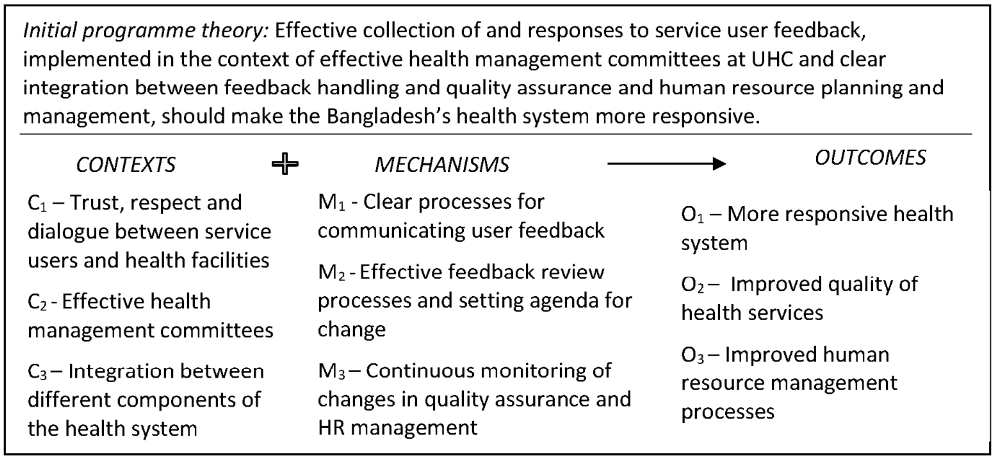

Figure 1: Conceptual Framework for Study

165x76mm (300 x 300 DPI)

For peer review only

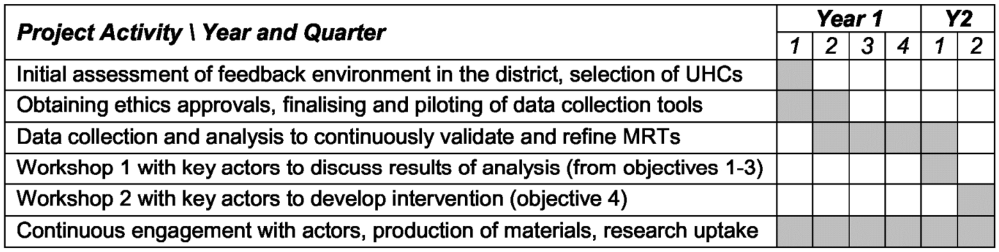

Figure 2: Project Workplan

163x40mm (300 x 300 DPI)

For peer review only

RESPOND project
Generic Guide for Interviews for Phase 1

Potential respondents include:
1. Residential Medical Officer or Upazila Health and Family Planning Officer (UHFPO)
2. Doctor (or paramedic) who has extensive knowledge of patients
3. A member of the management committee (who is not a staff at Upazila Health Complex)
4. A representative of Health Economic Unit or from UNICEF
5. Services users

This generic guide should be customized for the above groups of respondents.

Guidance notes
Before the interview
- Specify how long IDIs will take. You should familiarize yourself with the question guide and mark priority questions for the interview on the interview guide accordingly.
- You should also be familiar with the definitions and the terminology used in the RESPOND project glossary. You should be ready to clarify certain concepts and what they mean to the respondents during the interview
- As the interviews will be audio-recorded, you should check that the recorder is working properly before going into the interview.
- Check that you have notebooks and pens for note-taking and/or laptop computers as appropriate.
- Take extra copies participant information sheet, informed consent and other key documents that are deemed useful for each interview.

At the beginning of an interview
- Introduce yourself and other researchers present (if applicable) to the respondent.
- Briefly explain the objectives of the research and obtain informed consent.
- Check the overall time available for the interview.
- Explain that this will be an open interview and ascertain that respondent understand what is expected of them e.g. "In your own words, we would like you to tell us your personal knowledge and experiences of making use of user feedback systems in Bangladesh."

During the interview
- Although we are interested in the respondent's own views and experiences, for the purpose of RE, you are allowed to tactfully interrupt respondents to clarify issues related to the initial working theories or provide prompts. But do not try to always fill the silence with a next question or probe—your respondent may need some time to think about the issue.
- The bullet points in the next page provide a checklist of possible issues to raise. Use your judgement and ask these bullet points only if the conversation is faltering, or if the respondent is deviating too much from the question you asked them. Please note that some bullet points may not be applicable to some respondents.
- Keep track of the questions asked;
- Take note of non-verbal clues that might be important for analysis.
- If the interview is not being recorded (due to respondent refusal, or equipment failure), the note-taker's priority is to summarise the content of the interview
- During the interview, the interviewer should listen and be prepared to ask follow-up questions that further clarify issues.

For peer review only

- Use probes carefully; you do not need to ask all probes for all questions. Use your judgement and apply probes when the respondent is not answering fully or freely, or if the respondent doesn't appear to understand the question.
- Probe for specific examples from respondent's experience in support of her/his statements where possible.
- If the respondent is not answering a question in detail, ask 'give-me-an-example' type question to complement the question route.
- Always let the respondent finish a phrase; try not to interrupt her/him
- Use the interview guide flexibly, allowing the respondent to address the questions in any order but ensuring that the key issues are covered.

After the interview

- Thank the respondent/interviewee for their participation
- Remember to catalogue the tape/file (if you are recording)
- During debriefing, reflect on, discuss in the research team and clearly note down any other issues (e.g. respondent's mood during the pauses, your general impressions of the interview, good questions to ask) that may be important for future interviews, and the data analysis.
- Transcription if possible should start the day of the interview or at most the next day.

**Initial Working Theory (IWT)***	**Information to explore during the interview**	**Notes**
**IWT 1** *('trust and confidence to provide feedback'):* In the context of sufficient awareness of accessible channels for providing feedback (e.g. suggestion boxes, SMS etc.), coupled with the perception that the feedback system will not cause harm to them, and the existence of clear policy frameworks and Upazila management committees (MC) that provide timely and transparent responses on user feedbacks, these should increase service users' trust and confidence in the system and stimulate them to exercise their right to express their views and provide feedback about using established channels of feedback	1. What are the ways or systems through which patients can provide feedback about services they have used? *(probe on knowledge of different feedback systems)*<br>2. What motivates patients to provide/ prohibit feedback to UHCs, both positive and negative? E.g. ask, what made you complain in the first place? *(probe for perceptions regarding potential harm from feedback; hesitation to complain; awareness of right to complain)*<br>3. How do people / patients feel about the different feedback systems? *(probe for key pros and cons, e.g. ask about their experience of how long it takes the UHC to respond to and/or resolve their complaints)*	
IWT 2 *('valuing user views'):* In the context of clear policies and guidelines that accept the importance of user feedback for ensuring quality of care at Upazila level; coupled with a culture of documentation and communication of patient feedback; functional management committees (MC) and staff with relevant skills and knowledge; these should make health staff to feel supported (not threatened) and make them value and/ or respect	4. How do staff at UHC feel about patients communicating their feedback? *(probe on if/how they value feedback)*<br>5. What patient feedback channels are staff aware of? *(Probe for their reflections on their pros and cons?)*<br>6. How do staff feel about addressing patients' feedback? *(Do they feel supported, listened to, or do they feel threatened when they address such feedback?)*	

For peer review only

patients' views, leading to open transparent and discussion of patients feedback within existing quality improvement and human resource management frameworks at Upazila Health Complexes in Comilla district of Bangladesh.	7. How does this UHC deal with patient feedback and whether there is a link to QA and HRM? *(Probe for awareness of policy framework, views on transparency, accountability; the roles of MC or other structures / processes*?)	
IWT3 ('motivation to act upon feedback')<br>In the context where health staff in Comilla district are incentivized, supported and given adequate decision space to act upon patients feedback, coupled with availability of relevant policies, guides and processes that facilitate accountability, then health staff will feel safer about resolving issues raised by patients, value the processes and feel motivated to act upon the patients' feedback, thus leading to increased resolution of patient feedback, and provision of responses to patients; and increased patient satisfaction with the outcome of their feedback.	8. What structures, processes and systems exist at UHC to adequately deal with patient feedback? *(probe for decision space)*<br>9. What are the typical repercussions for staff for processing patient feedback? *(probe for sanctions/rewards and staff views on / feelings about them)*<br>10. What responses are usually provided to patients from the UHC, after resolving their feedback, if any? *(probe for mode of communication, and whether patients are satisfied with the outcome)*	

***Note:** Segments of IWTs 1-3 are coloured-coded to identify tentative Contexts (in blue ink), Mechanisms (in red ink) and Outcomes (in black ink), developed from sources including:

- researchers' understanding of the user feedback system
- informal engagements with UHC staff and key personnel from MOHFW Bangladesh
- review of relevant literature

For peer review only
