# Supplementary material for: Protocol for a mixed-methods realist evaluation of a health service user feedback system in Bangladesh
Source: BMJ Open. 2017 Jul 5;7(6):e017743. doi: 10.1136/bmjopen-2017-017743 (PMC5734574; doi:10.1136/bmjopen-2017-017743)
Supplement: Reviewer comments [file bmjopen-2017-017743.reviewer_comments.pdf]

PEER REVIEW HISTORY

BMJ Open publishes all reviews undertaken for accepted manuscripts. Reviewers are asked to complete a checklist review form (**http://bmjopen.bmj.com/site/about/resources/checklist.pdf**) and are provided with free text boxes to elaborate on their assessment. These free text comments are reproduced below.

ARTICLE DETAILS

**TITLE (PROVISIONAL)**	Protocol for a mixed methods realist evaluation of a health service user feedback system in Bangladesh.
**AUTHORS**	Ebenso, Bassey; Huque, Rumana; Azdi, Zunayed; Elsey, Helen; Nasreen, Shammi; Mirzoev, Tolib

This article was not externally reviewed at BMJ Open. Protocols that have been independently assessed prior to submission to BMJ Open are usually fast-tracked to publication on the grounds that further substantial changes will not be possible. This independent assessment will usually be external, independent review for both a substantial grant award from a non-commercial or government funder and ethics approval.
